# The chromosomal instability associated gene C2orf76 exerts dual roles in colorectal cancer through pyroptosis and chemotherapy response

**DOI:** 10.3389/fonc.2026.1866584

**Published:** 2026-08-12

**Authors:** Li Zhang, Zhu Song, Pinjie Zhang, Yuyu Jiang, Yan Xiang, Zeting Wang, Yingying Ding, Bing Rui, Yangyang Zhou, Junfeng Jiang, Shuqiang Cao, Xingguang Liu

**Affiliations:** 1 Department of Pathogen Biology, Naval Medical University, Shanghai, China; 2 Department of Pathology, Faculty of Medical lmaging, Naval Medical University, Shanghai, China; 3 School of Gongli Hospital Medical Technology, University of Shanghai for Science and Technology, Shanghai, China; 4 Department of Histology and Embryology, College of Basic Medicine, Naval Medical University, Shanghai, China

**Keywords:** C2orf76, chemotherapy response, chromosomal instability, colorectal cancer, pyroptosis

## Abstract

**Introduction:**

Despite advances in colorectal cancer (CRC) treatment, the chromosomal instability (CIN)-positive subgroup remains refractory to conventional therapies and immunotherapy. C2orf76, a poorly characterized gene identified as a CIN-linked candidate in CRC through bioinformatic analyses, has been associated with favorable prognosis and potential immune-related functions. However, its biological roles and underlying mechanisms in CRC remain largely unexplored.

**Methods:**

To investigate the role of C2orf76, we generated knockout (KO), overexpression (OE), and rescue (RE) cell lines in CRC models. We performed RNA-seq, RT-qPCR, and Western blotting to examine transcriptomic and protein-level changes. Functional assays included proliferation, migration, cisplatin sensitivity, pyroptosis (GSDME cleavage), cytokinesis-block micronucleus assay (CBMN) for CIN, and γ-H2AX foci detection for DNA double-strand breaks.

**Results:**

Unexpectedly, C2orf76 KO suppressed proliferation and migration, contradicting its favorable prognostic association and suggesting a complex regulatory role. RNA-seq revealed altered genes involved in chromosome segregation, cell cycle, platinum resistance, P53 signaling, and oncogenic pathways. RT-qPCR validated changes in chromosomal stability-related genes CDT1 and FOXM1, which were further verified by Western blotting. Further experiments showed that KO inhibited cisplatin-induced pyroptosis and cleavage of GSDME, whereas OE promoted migration and enhanced cisplatin sensitivity. The CBMN assay confirmed that C2orf76 significantly impacts chromosomal instability levels in CRC cells. C2orf76 KO cells also exhibited elevated γ-H2AX foci after cisplatin treatment. Rescue of C2orf76 expression reversed the KO-induced suppression of proliferation and migration, restored cisplatin sensitivity and pyroptosis, and reduced chromosomal instability.

**Discussion:**

These findings unveil a context-dependent duality of C2orf76: knockout increases CIN, reduces proliferation and migration, but promotes chemoresistance; overexpression enhances cisplatin-induced pyroptosis and drug sensitivity yet increases migration. Thus, C2orf76 establishes a functional link between CIN, cell death, and therapeutic response, positioning it as a tunable target that may require context-dependent modulation for effective CRC intervention. This study not only characterizes a previously understudied gene but also provides a mechanistic framework for understanding how CIN-associated genes can exert opposing effects on tumor progression and treatment outcome.

## 1 Introduction

Colorectal cancer is a common malignancy of the digestive tract, with global incidence projected to reach 2.2 million new cases and 1.1 million deaths by 2030 (1, 2). Current treatment modalities include surgery, radio- and chemotherapy, targeted agents, and immunotherapy (3). Despite advances, treatment resistance remains a major hurdle. Notably, chromosomal instability has been associated with poor therapeutic outcomes and immune evasion in several cancers (4, 5). Therefore, exploring CIN-associated genes in CRC may offer a basis for understanding resistance mechanisms and developing more effective interventions.

Genomic instability plays a critical role in predicting a patient’s potential response to tumor treatment. Microsatellite instability-high (MSI-H) is increasingly being recognized as the most important biomarker for identifying patients likely to benefit from immune checkpoint inhibitor therapy (3). Compared to MSI-H tumors, microsatellite stable (MSS) CRCs exhibit distinct molecular profiles, including lower levels of pyroptosis, a form of inflammatory cell death (6). Importantly, MSS CRCs can be further stratified into high chromosomal instability (CIN-high) and low chromosomal instability (CIN-low) subgroups based on the degree of chromosomal instability, with CIN-low patients demonstrating comparable survival rates and disease control rates to those with MSI-H CRC (7). High levels of CIN are generally associated with low immune scores and poor response to immunotherapy in cancer. Therefore, comparing the differentially expressed genes (DEGs) between these two patient groups will help to gain deeper insights into the underlying mechanisms and identify therapeutic targets.

Through bioinformatic analysis of public sequencing datasets from CRC patients with varying genomic instability, we identified C2orf76 as a key candidate, which not only showed differential expression between high- and low-CIN tumors but also exhibited prognostic significance. Furthermore, C2orf76 expression was suggested to have a potential association with immunotherapy response and tumor immune cell infiltration. However, C2orf76 (chromosome 2 open reading frame 76) remains largely uncharacterized, with only three reports to date (8–10). It was classified as a risk gene in HER2+ breast cancer in one multi-omics study (8), and was negatively correlated with RUNX3 (which regulates monocyte differentiation and inflammatory responses) in another (9). These findings point to a role for C2orf76 in cancer and immunity, consistent with our bioinformatic observations, but its function in colorectal cancer remains to be elucidated.

Here, we uncover a context-dependent duality of C2orf76 in colorectal cancer: knockout suppresses proliferation and migration, yet confers resistance to pyroptosis and elevates chromosomal instability—all of which are reversed by rescue—whereas overexpression promotes migration and enhances cisplatin sensitivity. These findings resolve the apparent paradox between its oncogenic activity *in vitro* and its favorable prognostic association. RNA-seq links C2orf76 to pathways regulating chromosomal integrity, cell cycle, platinum resistance, and p53 signaling. Thus, C2orf76 emerges as a candidate target for therapeutic strategies that leverage pyroptosis to elicit anti-tumor immunity and overcome chemoresistance.

## 2 Materials and methods

### 2.1 Data collection and bioinformatic analyses

Data of CRC patients with different level of genome instability (GSE30540) were collected from GEO datasets (https://www.ncbi.nlm.nih.gov/geo/). Differentially expressed genes between CIN-high and CIN-low patients were obtained and filtered to subsequent analysis. The survival rate of CRC patients with or without TP53 mutation was analyzed by Kaplan-Meier curves using The Kaplan Meier plotter platform (www.kmplot.com/). Expression and prioritization by clinical association to immune therapy were carried out on GEPIA (http://gepia.cancer-pku.cn/) and the CIDE (https://cide.ccr.cancer.gov/) platform. The correlation between DEG and cancer immune infiltrates was analyzed by TIMER 3 (https://compbio.cn/timer3/). Validation of the differential expression of C2orf76 in colorectal cancer patients with varying levels of chromosomal instability was performed using dataset GSE-34489 and the cBioPortal platform (https://www.cbioportal.org/).

### 2.2 Cell culture and transfection

Colorectal cancer cell lines HCT-116 and SW480 were cultured in DMEM with 10% FBS and penicillin-streptomycin. Cells were maintained as monolayer cultures at 37 °C in a humidified atmosphere containing 5% CO_2_.

Cells were cultured for 24 h prior to transfection and were then transfected with the corresponding vector using Lipofectamine 3000 Transfection Reagent (Invitrogen, Carlsbad, CA, USA) according to the manufacturer’s instructions. For the overexpression of C2orf76, recombinant pGC-LV-C2orf76-GFP vectors containing either C2orf76 mRNA or a scrambled control sequence were constructed by GENEWIZ Company (China). C2orf76 knockout cells were rescued by stable transfection of a vector expressing a synonymous-mutated C2orf76 transcript (GENEWIZ, China). Lentivirus-mediated infection of cells was performed according to the manufacturer’s recommended MOI value. Transfection efficiency was validated by Western blot. C2orf76, TP53, and FOXM1 siRNA were purchased from GenePharma (China). Lipofectamine^®^ RNA iMAX Reagent was used for siRNA transfection assays as direction of manual handbook.

### 2.3 CRISPR/Cas9 approach to generate C2orf76 knockout HCT-116 clones

C2orf76 knockout clones were generated using a two-step CRISPR/Cas9 approach in HCT-116 cells with C2orf76-targeting and non-targeting control synthetic guide RNAs (sgRNAs) according to the manufacturer (OBiO Technology, China). Briefly, HCT-116 cells were transduced with lentivirus particles containing two independent C2orf76 sgRNAs or a NT-Control that co-expressed blue fluorescent protein (BFP). Cells were sorted (BFP+) by fluorescence-activated cell sorting (FACS), with successfully transduced populations transiently transfected with a Cas9 and green fluorescent protein (GFP) expression plasmid. FACS was used to recover BFP+/GFP+ cells, with individual clones isolated through subsequent serial dilutions. Putative C2orf76 knockout clones (Clone G6) were identified by western blot, with DNA sequencing analysis used to identify the allele-specific edits.

### 2.4 Western blotting

Total protein was isolated from cells using ice-cold radio immunoprecipitation assay buffer and quantified with the BCA protein assay reagent. Equal amounts of protein were separated by SDS-PAGE and transferred to polyvinylidene fluoride membranes. After blocking with 5% milk for 1 h, the membranes were incubated with primary antibodies overnight at 4 °C. The signals were then detected after washing and incubation with secondary antibodies using ECL Western blotting detection reagents. The antibodies used in this study were as follows: β-actin (Proteintech, 15526-1-AP), C2orf76 (Immunoway, YN4538), CDT1 (Abcam, ab202067), FOXM1 (Ab-mart, TD6962) and Gasdermin E (GSDME) (Abcam, ab215191).

### 2.5 Clonogenic assay

Clonogenic growth was assessed by seeding C2orf76 knockout (KO), overexpression (OE), rescue (RE), and corresponding empty-vector control clones at low density (800 cells/well) into six-well plates. Cells were grown for 14 days, at which point they were fixed (4% paraformaldehyde), stained (0.005% crystal violet) and scanned using a HP Officejet 4620 series scanner. Images were imported into ImageJ for processing and analysis in which colony numbers and sizes were automatically determined. Colonies were operationally defined as those with a diameter ≥ 100 μm in size.

### 2.6 Cell viability

CCK8 assay was used to analyze cell viability (CCK-8 Kit; MedChemExpress, New Jersey, USA). Cell lines were counted, seeded into 96-well plate with 100 µl normal culture medium at 37°C. At the indicated time-points (0 h, 24 h, 48 h and 72 h), 10 µl of CCK-8 solution was added into each well and then cultured for 1 h at 37°C. The absorbance of each well at 450 nm (A450) was detected with an BioTek Epoch (BioTek Instruments, Vermont, USA).

### 2.7 Wound healing assay

For wound healing assays, stably transfected cells were seeded into 6-well plates, and an artificial wound was created using a 10-µL pipette tip when the cells reached 90% confluence. The cells were then cultured in medium containing 1% FBS for 24 h. Images were taken to record the wound width at 0h, 24h and 48h. Cell migration ability was evaluated by comparing the wound widths of different groups at 24h and 48h.

### 2.8 Transwell migration assay

Cell migration was assessed using 24-well Transwell chambers (Corning, USA) equipped with polycarbonate membrane inserts (8-μm pore size). SW480 cells with C2orf76 intervention were serum-starved overnight, and 1 × 10^5^ cells suspended in 200 μL of serum-free medium were seeded into the upper chamber. The lower chamber was filled with 600 μL of complete medium containing 10% fetal bovine serum (FBS) as a chemoattractant. After incubation at 37 °C with 5% CO_2_ for 36h, non-migrated cells on the upper surface of the membrane were gently removed with a cotton swab. The migrated cells attached to the lower surface were fixed with 4% paraformaldehyde and stained with 0.1% crystal violet. For quantification, stained cells were photographed under an inverted microscope (100× magnification), and five random fields per insert were counted.

### 2.9 Drug tolerance assay

HCT116 cell lines with C2orf76 intervention were seeded in 96-well plates at 1 × 10^4^ cells per well and allowed to adhere overnight. The following day, cells were treated with cisplatin at concentrations of 5, 10, or 20 μg/mL. After 12, 24, 36, or 48 h of treatment, cell viability was assessed using the Cell Counting Kit-8 (MCE, USA) according to the manufacturer’s instructions. Absorbance was measured at 450 nm using a microplate reader. Relative cell viability was calculated by normalizing to untreated control cells at each time point. For oxaliplatin and irinotecan treatments, HCT116 cells with C2orf76 intervention were treated with oxaliplatin (100 μM) or irinotecan (20 μM) for 48 h. The concentrations and duration were chosen based on the manufacturers’ instructions.

### 2.10 Flow cytometry assay

After undergoing the treatment of Cisplatin (10μg/mL, 36 h), cells were trypsinized (excluding EGTA/EDTA), harvested while retaining all floating cells, and subsequently washed with PBS. These cells were then stained with YO-PRO-1, following the guidelines provided by Kit Manufacturer (C2022; Beyotime, Shanghai, China). Finally, the stained samples were analyzed using flow cytometry.

### 2.11 Lactate dehydrogenase release assay

Cells were evenly seeded into 96-well plates at a density of 1.0×10^4^ cells per well and treated with specific interventions. The assay solution was prepared according to the standardized procedure outlined in the kit instructions (LDH Release Assay Kit with WST-8, C0019S, Beyotime Biotechnology, China) and mixed with the cell supernatant. The mixture was then incubated in the dark for 30 min. Optical density values were then measured at 450 nm. Each sample was assessed thrice to determine the actual lactate dehydrogenase (LDH) release.

### 2.12 Live/dead assay

Cell viability was evaluated through live/dead staining. The cells were cultured for up to 48 h, after which the medium was removed and cells were treated with cisplatin at 10 µg/mL for 36 h. After treatment, cells were incubated in pre-warmed culture medium containing either Hoechst 33342 (10 µg/mL) or DAPI (1 µg/mL) for nuclear counterstaining, together with propidium iodide (PI, 50 µg/mL) and YO-PRO-1 (0.5 µM) to label dead or membrane-permeable cells. The staining solution was applied for 20 min at 37 °C in the dark. Following staining, cells were gently washed with PBS and immediately imaged using a fluorescence microscope. Hoechst 33342 or DAPI stained all nuclei (blue), PI labeled late apoptotic/necrotic cells (red), and YO-PRO-1 identified early apoptotic cells with increased membrane permeability (green). For quantification, at least five random fields per group were captured, and the percentages of PI-positive and YO-PRO-1-positive cells were calculated relative to the total number of nuclei. Each experiment was performed in triplicate.

### 2.13 Cytokinesis-block micronucleus assay

HCT116 cells with C2orf76 intervention were cultured in DMEM medium with 10% FBS and 1% penicillin–streptomycin at 37 °C with 5% CO_2_. Cells were seeded in 6-well plates at 2.5 × 10^5^ cells/well. After 24 h, the medium was replaced with fresh medium containing 6 µg/mL cytochalasin B (MCE) to block cytokinesis. Following 48 h of cytochalasin B exposure, cells were harvested by trypsinization, subjected to hypotonic treatment with 0.075 M KCl for 10 min at 37 °C, and fixed three times with methanol: acetic acid (3:1). Cell suspensions were dropped onto pre-cleaned glass slides, air-dried, and stained with DAPI.

For each experimental condition, at least 1000 binucleated cells were scored blindly under a light microscope (400× magnification). Micronuclei were identified as small, round structures within the cytoplasm of binucleated cells, with a diameter 1/16–1/3 of the main nuclei. Micronucleus frequency was calculated as the percentage of binucleated cells containing micronuclei.

### 2.14 Immunofluorescence staining for γ-H2AX

Immunofluorescence staining for γ-H2AX was performed using the DNA Damage Assay Kit by γ-H2AX Immunofluorescence (C2036s, Beyotime, China) according to the manufacturer’s instructions. Briefly, cells cultured on coverslips in 6-well plates were washed once with PBS and fixed with 4% paraformaldehyde for 5–15 min at room temperature. After three washes with washing buffer (5 min each), cells were blocked with blocking buffer for 10–20 min at room temperature. The cells were then incubated with anti-γ-H2AX rabbit monoclonal antibody (1 ml per well) overnight at 4 °C or for 1 h at room temperature. After three washes (5–10 min each), the cells were incubated with Cy3-conjugated anti-rabbit secondary antibody (1 ml per well) for 1 h at room temperature in the dark. Following two additional washes (5–10 min each), nuclei were counterstained with DAPI (1 ml per well) for 5 min. After three final washes (3–5 min each), the coverslips were mounted with antifade mounting medium and examined under a fluorescence microscope. γ-H2AX appeared as red foci, while nuclei were stained blue (DAPI).

### 2.15 RNA-sequence

HCT116 wild-type and C2orf76-knockout cells (3 *vs* 3) were cultured in DMEM supplemented with 10% fetal bovine serum and 1% penicillin–streptomycin at 37 °C in a 5% CO_2_ atmosphere. Cells in the logarithmic growth phase (70–90% confluence) were harvested for RNA extraction. RNA-seq was performed with three biological replicates per cell line. Total RNA was isolated and the quantity and purity of RNA were assessed using a NanoDrop spectrophotometer, and RNA integrity was evaluated with an Agilent 2100 Bioanalyzer. Only samples with A260/A280 ratios between 1.8 and 2.0 and RNA integrity number (RIN) values ≥ 7.0 were used for subsequent library preparation. A total of 3 × 10^6^ to 5 × 10^6^ cells were used per sample to obtain at least 1 µg of total RNA.

The Hieff NGS^®^ Ultima Dual mode mRNA Library Prep Kit was used to construct RNA-seq libraries according to the manufacturer’s instructions. High-quality clean reads were further filtered by fastp (version 0.18.0). Pair-end clean reads were aligned to the ensemble-release 113 human genome using the HISAT2 (v2.1.0) with default parameters (**Supplementary Figure 1**). For each protein coding gene, a TPM (Transcripts Per Kilobase of exon model per Million mapped reads) value was calculated to quantify its expression abundance using RSEM software. Differentially expressed genes (DEGs) were identified using DESeq2 with a threshold of | log_2_(fold change) | > 1 and an adjusted p-value (FDR) < 0.05.

### 2.16 Real-time quantitative PCR

Total RNA was isolated from cell lines using Steady Pure Universal RNA Extraction Kit II (Accurate Biotechnology, Hunan, China). Two µg of RNA was reverse transcribed into cDNA using HiScript III All-in-one RT SuperMix (Vazyme, Jiangsu, China). Quantitative PCR was carried out using SupReal Q Purple Universal SYBR qPCR Master Mix (Vazyme, Jiangsu, China) with a final volume of 20 μL and using the Heal Force X960 Real-Time PCR System (Heal Force Bio-Meditech, Shanghai, China). Target mRNA expression was normalized to GAPDH. **Supplementary Table 1** shows the detailed sequences of the PCR primers included in this study.

### 2.17 Statistical analysis

All experiments were performed in triplicate independently (n = 3). Student’s t-test and two-way ANOVA were used in statistical analyses and performed by GraphPad Prism. Data were presented as mean ± SEM. A P value of 0.05 or less were considered to be significant. In all figures, the level of significance is reported as follows: NS > 0.05, *P < 0.05, **P < 0.01, ***P < 0.001, and ****P < 0.0001.

## 3 Results

### 3.1 C2orf76 is associated with survival and immune response in patients with CRC

**Figure 1:**
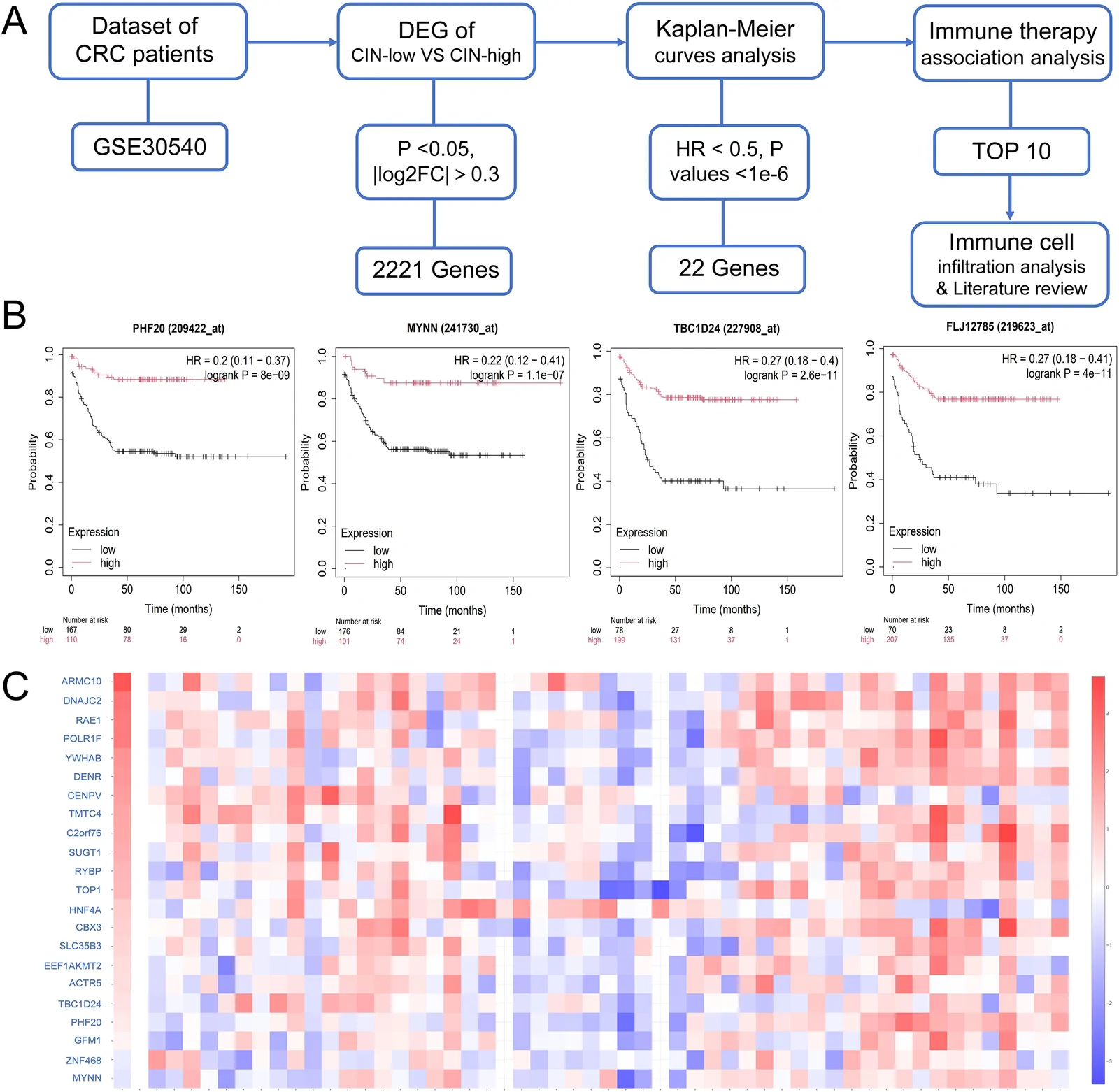
Screening and integrated bioinformatic analysis of CIN associated DEGs. **(A)** Schematic illustration of gene selection, data processing, and analysis of CIN associated DEGs (GSE30540). **(B)** DEGs analyzed by Kaplan-Meier curves using The Kaplan Meier plotter platform (www.kmplot.com). The top 4 genes are shown. **(C)** Prioritization of the 22 genes (filtered by Kaplan-Meier survival analysis) based on their clinical association with tumor immunotherapy, performed using the CIDE platform (https://cide.ccr.cancer.gov/).

**Figure 2:**
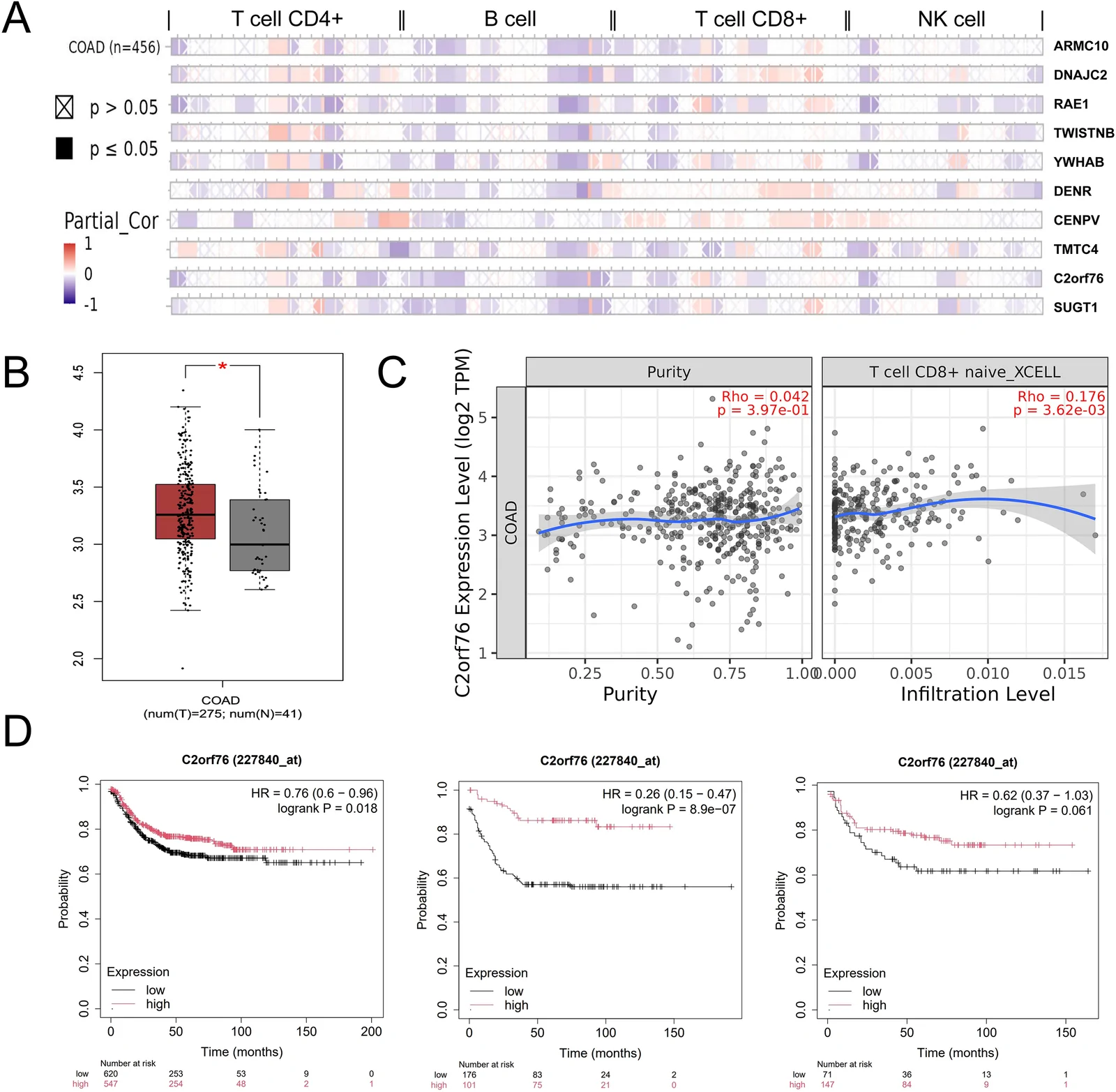
Integrated bioinformatic analysis of CIN associated C2orf76 in CRC. **(A)** Analysis of the correlation between expression of the top 10 genes (prioritized by the CIDE platform) and immune cell infiltration in tumors, performed using the TIMER 3 database (https://compbio.cn/timer3/). **(B)** Expression of C2orf76 in colorectal cancer based on bioinformatics analysis using the GEPIA platform (http://gepia.cancer-pku.cn/). **(C)** Analysis of the correlation between C2orf76 expression and CD8+ T-cell infiltration in tumors using the TIMER 3 database (https://compbio.cn/timer3/). **(D)** Kaplan-Meier survival analysis of colorectal cancer patients based on C2orf76 expression, stratified by TP53 mutation status (Left: all patients; Middle: TP53-mutant; Right: TP53-wild-type), generated using the Kaplan-Meier plotter platform (www.kmplot.com). *p < 0.05, **p < 0.01, ***p < 0.001, ****p < 0.0001; NS, not significant.

Given that chromosomal instability drives therapy resistance, immune evasion, metastasis, and poor prognosis (11–15), we screened DEGs between CIN-high and CIN-low CRC patients using the GSE30540 dataset (**Figure 1A**). Among 2, 221 DEGs (p<0.05, |log_2_FC|>0.3) (**Supplementary Table 2**), we performed Kaplan-Meier analysis (KM-Plotter) (**Figure 1B**; **Supplementary Figure 2**). Given that TP53 dysfunction promotes CIN tolerance and synergistically drives malignant tumor progression (16–18), we further analyzed the correlation between candidate gene expression and prognosis in patients with TP53 mutations. Twenty-two genes most impacting survival (HR<0.5, p<1e-6) were prioritized based on immunotherapy relevance (**Figure 1C**). Immune infiltration analysis and literature review focused on the top 10 candidates (**Figure 2A**). Among five immune infiltration-associated genes, C2orf76 attracted attention as only three publications have reported it, indicating limited knowledge of its role in tumors. As shown in **Figure 2B**, C2orf76 was upregulated in colon adenocarcinoma tissues versus normal tissues. Moreover, high C2orf76 expression correlated with better survival, especially in TP53-mutant patients (**Figure 2D**).

To validate C2orf76 expression in CRC patients with different CIN levels, we used the validation set (GSE-34489) from the original study (derived from GSE14333) and obtained consistent results (**Supplementary Table 3**). Subgroup analysis in cBioPortal by CIN subtypes showed significant differential expression of C2orf76 in the CIN subtype versus both GS (**Supplementary Table 4**) and MSI subtypes (**Supplementary Table 5**). TIMER analysis revealed potential correlations between C2orf76 and immune cell infiltration (**Figure 2C**; **Supplementary Figure 3**). Among pro-tumor immune cells, Tregs negatively correlate with C2orf76, while M2 macrophages and MDSCs positively correlate. Among anti-tumor cells, activated CD8^+^ T cells and dendritic cells show positive correlation, whereas M1 macrophages and NK cells show negative correlation. Given the low immune cell specificity of C2orf76 (Human Protein Atlas, **Supplementary Figure 4**), its overall effect on tumor immune infiltration is confounding and requires further validation.

### 3.2 Knockout of C2orf76 suppresses the proliferation and migration of colorectal cancer cells

**Figure 3:**
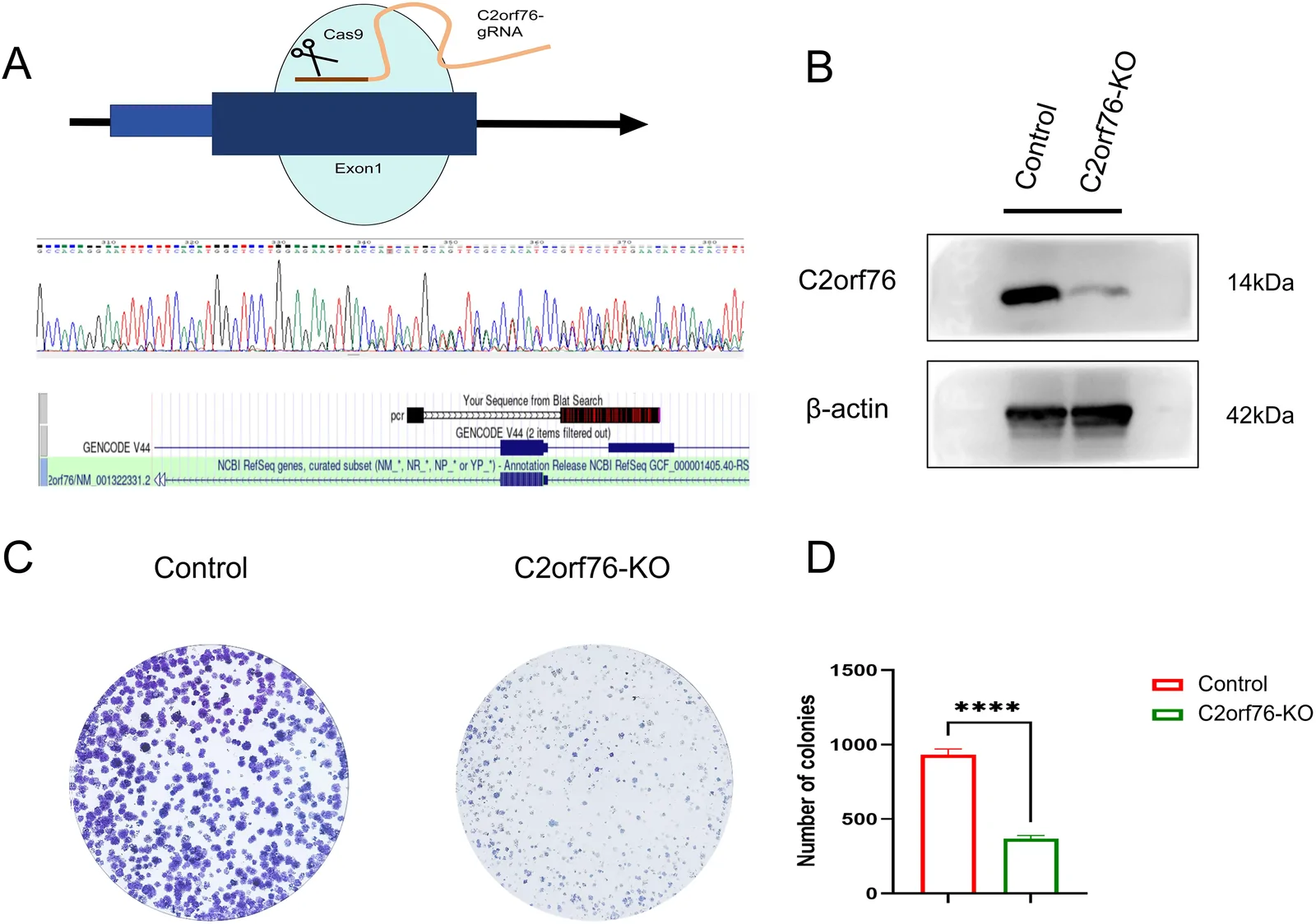
Establishment and functional validation of C2orf76-KO HCT116 cells. **(A)** C2orf76 knockout cell line was generated in HCT116 cells using CRISPR-Cas9, and successful gene disruption was confirmed by Sanger sequencing of monoclonal populations. **(B)** Knockout of C2orf76 in CRC cell was validated by western blot. **(C)** Representative images of crystal violet-stained colonies from wild-type and C2orf76-KO HCT116 cells. **(D)** Quantification of colony numbers (n ≥ 3). C2orf76 knockout significantly suppressed colony formation. *p < 0.05, **p < 0.01, ***p < 0.001, ****p < 0.0001; NS, not significant.

**Figure 4:**
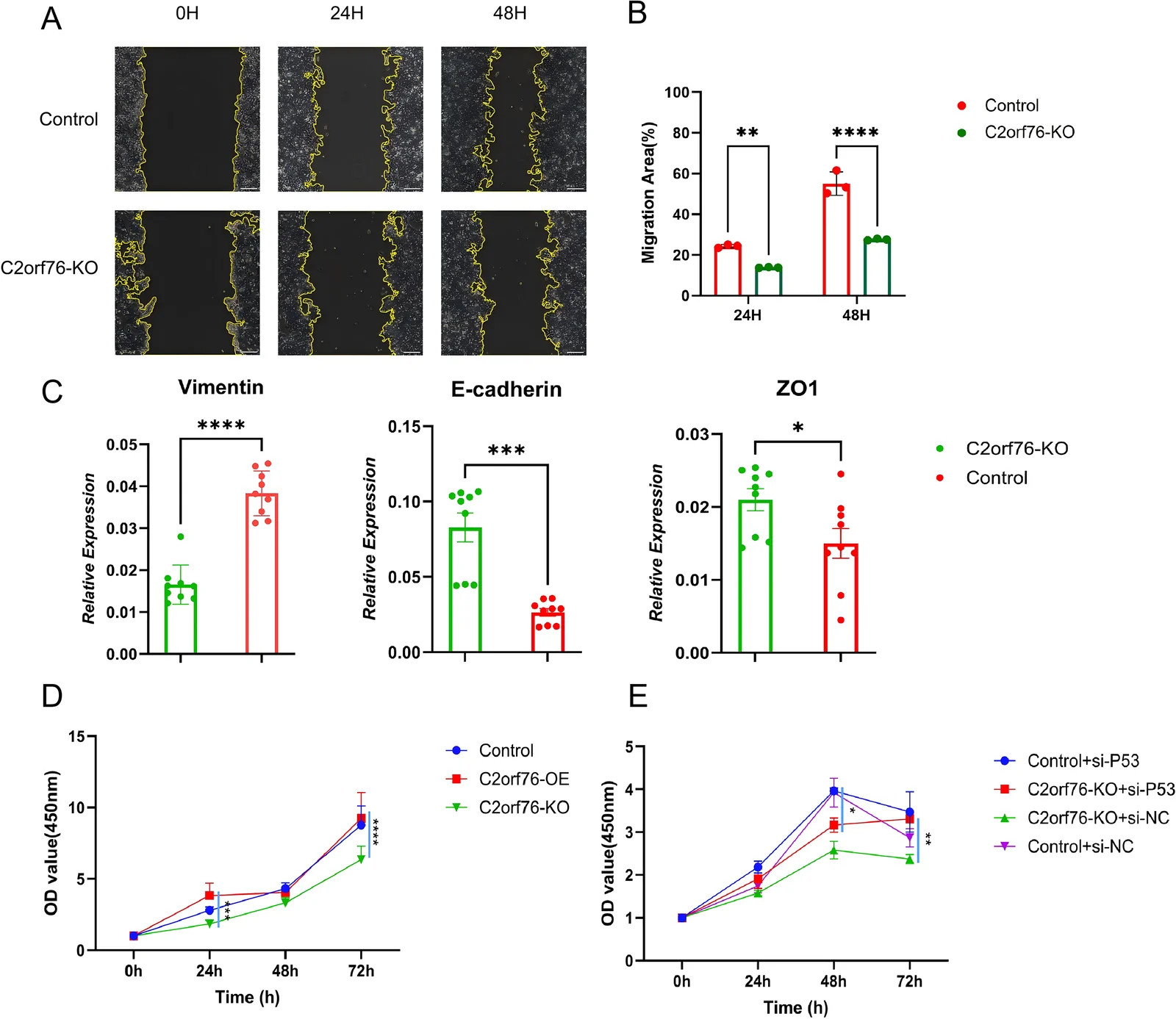
Modulation of C2orf76 expression affects CRC cell viability and migration. **(A, B)** Wound healing assay. A scratch was created in confluent wild-type and C2orf76-KO cell monolayers, and closure was monitored at 0, 24, and 48 h. Representative images **(A)** and quantified wound closure **(B)** are shown. KO significantly reduced migration versus wild-type controls. Scale bar, 50 μm. **(C)** qPCR analysis of migration-related marker genes in C2orf76-KO and control HCT116 cells. KO cells showed significant changes in the expression of Vimentin, E-cadherin, and ZO1 relative to controls, indicating reduced migratory capacity. **(D)** CCK-8 viability assay. Control, C2orf76-overexpressing (OE), and C2orf76-knockout (KO) cells were seeded in 96-well plates, and OD450 was recorded daily for 3 days. KO markedly decreased viability from day 1 onward. **(E)** CCK‑8 proliferation assay. Wild‑type (Control) and C2orf76‑knockout (KO) cells were transfected with TP53‑siRNA or NC‑siRNA, and absorbance at 450 nm was recorded daily for 3 days. TP53 knockdown partially rescued the viability defect in KO cells but had little effect on wild-type control cells. *p < 0.05, **p < 0.01, ***p < 0.001, ****p < 0.0001; NS, not significant.

To investigate C2orf76 function in colorectal cancer, we generated a stable C2orf76-knockout HCT116 cell line using CRISPR-Cas9 (**Figure 3A**) and confirmed protein depletion by Western blotting (**Figure 3B**). Colony formation assays demonstrated that knockout significantly impaired proliferation (**Figures 3C, D**). Wound healing assays revealed reduced migration in KO cells (**Figure 4A, B**). Real‑time quantitative PCR was performed to assess the expression of migration marker genes (Vimentin, E‑cadherin, and ZO1) in colorectal cancer cells (**Figure 4C**). C2orf76 knockout markedly affected all three markers, consistent with the altered migration observed in wound healing assays, further supporting a role for C2orf76 in cell migration. CCK‑8 assays further confirmed that knockout significantly impaired proliferation (**Figure 4D**). These results indicate that C2orf76 critically regulates CRC cell proliferation and migration. Given the prognostic interaction between C2orf76 and TP53, we transfected TP53 siRNA into both knockout and wild‑type HCT116 cells and assessed proliferation by CCK‑8. TP53 knockdown partially restored proliferation in KO cells at 48 h, with maximal rescue at 72 h and statistical significance versus KO controls (**Figure 4E**).

### 3.3 C2orf76 knockout is associated with chromosome status and related gene expression

**Figure 5:**
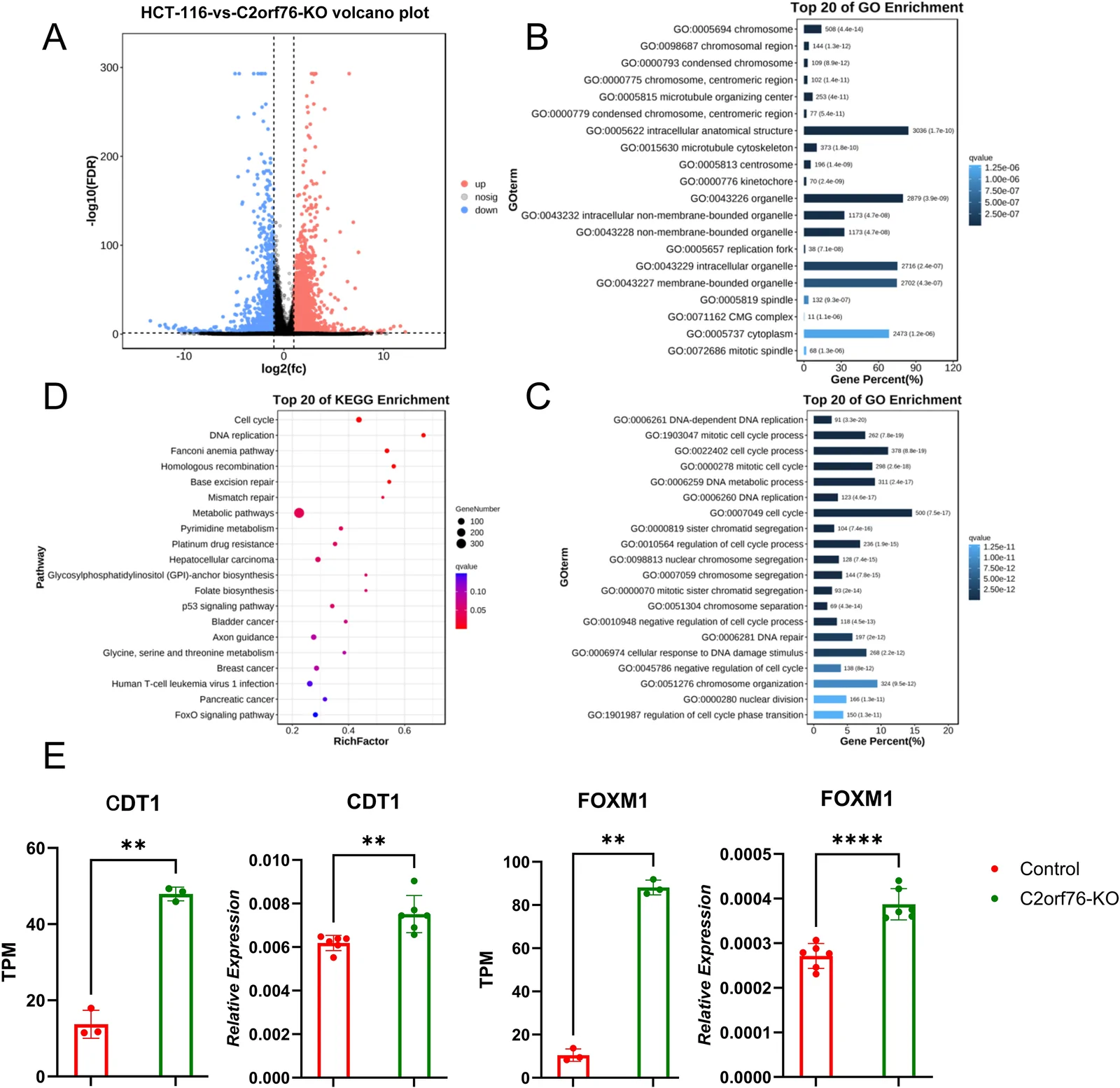
RNA-sequencing to analyze the correlation between C2orf76 and the biological function of colorectal cancer cells. **(A)** Volcano plot of genes differentially expressed in C2orf76 knockout and wild-type CRC cells. **(B)** GO enrichment analysis of Cellular Component for significant DEGs after C2orf76 knockout. **(C)** GO enrichment analysis of Biological Process for significant DEGs after C2orf76 knockout. **(D)** KEGG enrichment analysis for significant DEGs after C2orf76 knockout. **(E)** Validation of C2orf76 correlation with CDT1 and FOXM1 in CRC cells via RT-qPCR. *p < 0.05, **p < 0.01, ***p < 0.001, ****p < 0.0001; NS, not significant.

**Figure 6:**
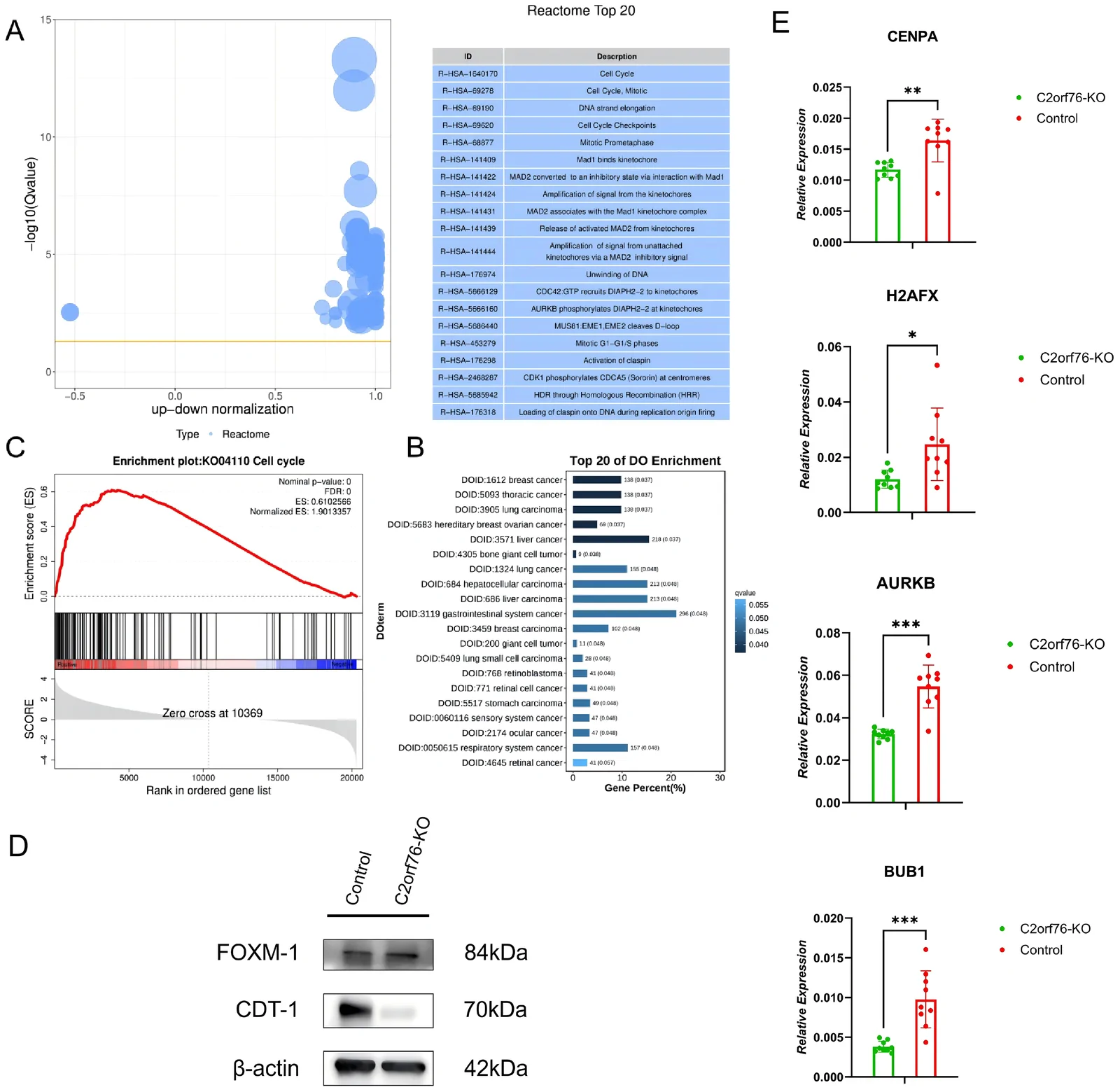
Enrichment analyses of DEGs after C2orf76 knockout and validation of key genes. **(A)** Reactome pathway bubble plot shows most top 20 significant Reactome pathway. **(B)** DO enrichment analysis for significant DEGs after C2orf76 knockout. **(C)** KEGG pathway enrichment analysis by GSEA. **(D)** Western blot validation: FOXM1 protein is upregulated (consistent with mRNA), whereas CDT1 protein is downregulated (opposite to its mRNA change). **(E)** qPCR detection of chromosome instability-related genes CENPA, H2AFX, AURKB, and BUB1, all showing significant downregulation. Data represent at least three independent experiments. *p < 0.05, **p < 0.01, ***p < 0.001, ****p < 0.0001; NS, not significant.

RNA-seq of C2orf76 knockout cells identified 3,816 DEGs (2,630 up, 1,186 down) (**Figure 5A**). GO Cellular Component analysis revealed that the DEGs were significantly enriched in chromosome associated regions (**Figure 5B**). GO Biological Process indicated enrichment in cell cycle, chromosome segregation, DNA replication/repair, suggesting C2orf76 influences chromosomal and genome stability (**Figure 5C**). KEGG analysis confirmed effects on cell cycle, DNA repair and homologous recombination. The platinum resistance and TP53 pathway, which emerged from the KEGG analysis, may account for the observed survival differences (**Figure 5D**; **Supplementary Figure 5**). Disease Ontology (DO) and Reactome enrichment analyses further confirmed the prominent role of C2orf76 in cancer and cell cycle-related pathways (**Figures 6A, B**). Subsequent Gene Set Enrichment Analysis (GSEA) of KEGG pathways, using a weighted statistic to rank genes, revealed significantly affected pathways, with the top 20 core enrichment genes listed (**Figure 6C**; **Supplementary Figure 6**). All GO and KEGG analyses used full DEGs.

Consistently, CDT1 and FOXM1—key drivers of genomic and chromosomal instability—showed negative correlation with C2orf76 expression in both sequencing data and RT-qPCR validation (**Figure 5E**). Western blot analysis further confirmed that FOXM1 protein levels were upregulated upon C2orf76 knockout. In contrast, CDT1 protein expression was markedly reduced in KO cells, which differed from its RNA-level trend (**Figure 6D**). To explore this discrepancy, we additionally examined the RNA expression of several other chromosome stability-related genes, including AURKB, BUB1, CENPA, and H2AFX (encoding γ-H2AX). All four genes showed decreased mRNA levels following C2orf76 deletion, further supporting the role of C2orf76 in affecting chromosomal stability (**Figure 6E**).

### 3.4 Knockout of C2orf76 confers resistance to cisplatin and pyroptosis in CRC cells

**Figure 7:**
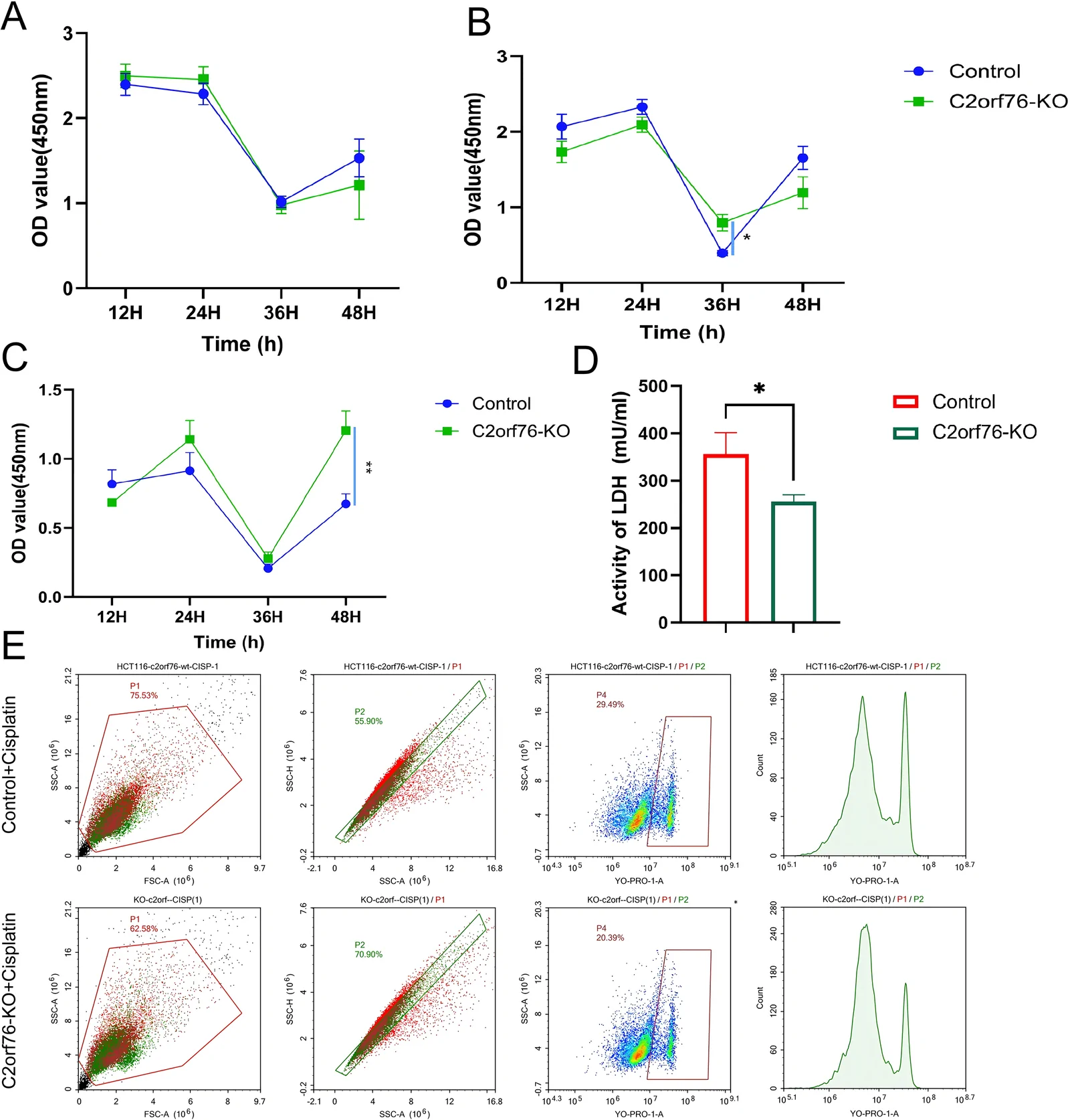
C2orf76 knockout confers resistance to cisplatin in CRC cells. **(A-C)** CCK-8 assay of cisplatin-treated C2orf76 knockout and wild-type control CRC cells. Cells were exposed to 5 μg/mL **(A)**, 10 μg/mL **(B)**, or 20 μg/mL **(C)** cisplatin for 12, 24, 36, and 48 hours. Optimal conditions were achieved at a concentration of 10 μg/mL and an incubation period of 36 h. **(D)** Lactate dehydrogenase (LDH) release assay showed that after cisplatin treatment (10 μg/mL, 36 h), LDH release was significantly reduced in C2orf76-knockout (KO) cells compared with wild-type control cells. **(E)** Flow cytometric analysis using YO-PRO-1 staining was performed on cisplatin-treated wild-type and C2orf76-knockout (KO) CRC cells. KO cells exhibited a lower percentage of YO-PRO-1-positive cells than WT controls, indicating reduced sensitivity to cisplatin. *p < 0.05, **p < 0.01, ***p < 0.001, ****p < 0.0001; NS, not significant.

To validate RNA-seq KEGG results linking C2orf76 to platinum resistance, we treated C2orf76-knockout and wild-type HCT116 cells with cisplatin (5, 10, 20 μg/mL) for 12–48 h (**Figures 7A-C**). The optimal condition (10 μg/mL, 36 h) showed lowest viability. LDH release assay confirmed C2orf76 knockout protected tumor cells: increased viability and decreased LDH release versus control (**Figure 7D**). Flow cytometry showed C2orf76 knockout attenuated cisplatin-induced cell death, with reduced quantifiable death population (**Figure 7E**).

**Figure 8:**
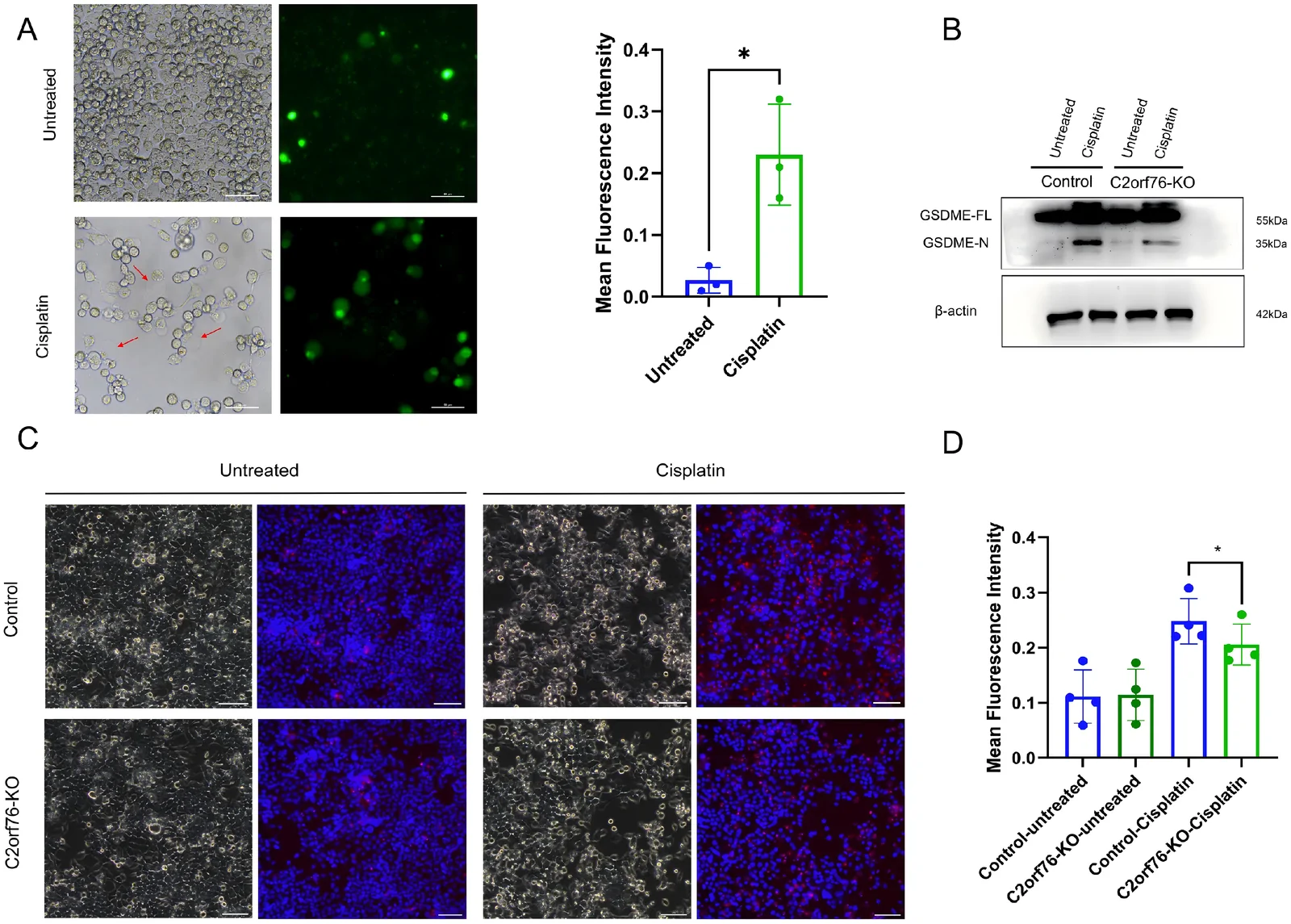
C2orf76 knockout confers resistance to pyroptosis in CRC cells. **(A)** Representative images and bar graphs showing the percentage of YO-PRO-1-positive pyroptotic cells in cisplatin-treated CRC cells. Scale bar, 50 μm. **(B)** Western blot analysis of GSDME expression in wild-type and C2orf76-knockout (KO) CRC cells with or without cisplatin treatment. KO cells showed attenuated cisplatin-induced GSDME cleavage, as evidenced by reduced GSDME-N fragment levels. **(C, D)** Live-dead cell staining (Hoechst 33342 for live cells, blue; propidium iodide for dead cells, red) of cisplatin-treated wild-type and KO cells. KO cells exhibited a significantly lower proportion of dead cells compared with wild-type controls, confirming reduced cell death. Scale bar, 50 μm. *p < 0.05, **p < 0.01, ***p < 0.001, **p < 0.0001; NS, not significant.

Cisplatin triggers diverse programmed cell deaths (apoptosis, autophagy, ferroptosis, pyroptosis), among which pyroptosis—a pro-inflammatory form that synergizes with immunotherapy—is defined by cellular swelling and membrane rupture, eliciting strong inflammation (19–22). In cisplatin-treated CRC cells, we observed typical pyroptotic morphology (**Figure 8A**). Since bioinformatics analyses suggested a potential link between C2orf76 and tumor immunity, we examined GSDME—the core pyroptosis executioner—in C2orf76-knockout cells by Western blot. In control cells, cisplatin promoted GSDME cleavage, confirming pyroptosis induction; however, C2orf76 knockout attenuated this cleavage, as evidenced by reduced GSDME-N fragment intensity (**Figure 8B**). Live-dead staining further confirmed decreased cell death after knockout (**Figures 8C, D**). Collectively, C2orf76 ablation attenuates pyroptosis and impairs the response to platinum-based chemotherapy (cisplatin) in colorectal cancer.

### 3.5 Overexpression and rescue of C2orf76 differentially regulate migration and chemosensitivity in CRC cells.

**Figure 9:**
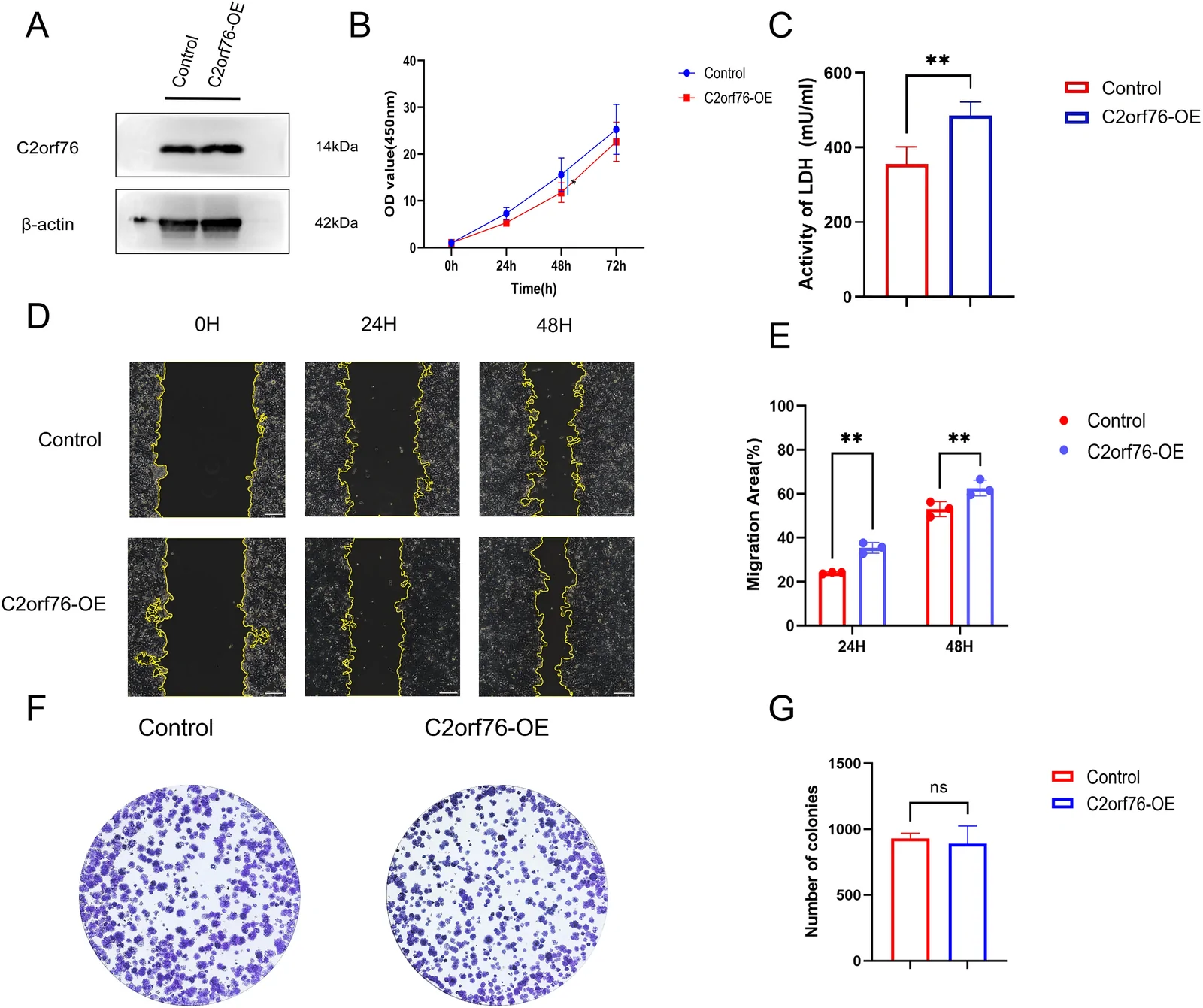
C2orf76 overexpression promotes migration and sensitizes CRC cells to cisplatin. **(A)** The construction of C2orf76 overexpression models in a CRC cell line was confirmed by western blot. **(B)** CCK-8 proliferation assay over a 72-h period. No significant difference was observed between OE and control cells, except at the 48-h time point. **(C)** LDH release assay evaluating cisplatin-induced cytotoxicity. OE cells exhibited increased LDH release compared with controls, indicating enhanced sensitivity to cisplatin. **(D, E)** Representative images **(D)** and quantitative analysis **(E)** of wound healing assays. OE cells showed significantly enhanced migratory capacity compared with controls. Scale bar, 50 μm. **(F, G)** Representative images **(F)** and quantitative analysis **(G)** of colony formation assays. OE cells formed colonies at a frequency comparable to that of controls, consistent with the proliferation results. *p < 0.05, **p < 0.01, ***p < 0.001, ****p < 0.0001; NS, not significant.

C2orf76-overexpressing (OE) HCT116 cells were generated by lentiviral transfection and validated via Western blot (**Figure 9A**). Except at the 48-hour time point, C2orf76 overexpression did not demonstrate a significant impact on CRC cell proliferation (**Figure 9B**), a finding consistent with the results from the colony formation assay (**Figures 9F, G**). In contrast, LDH assays revealed that C2orf76 overexpression sensitized colorectal cancer cells to the chemotherapeutic agent cisplatin (**Figure 9C**). Furthermore, the wound healing assay indicated that C2orf76 overexpression significantly enhanced the migratory capacity of colorectal cancer cells (**Figures 9D, E**).

**Figure 10:**
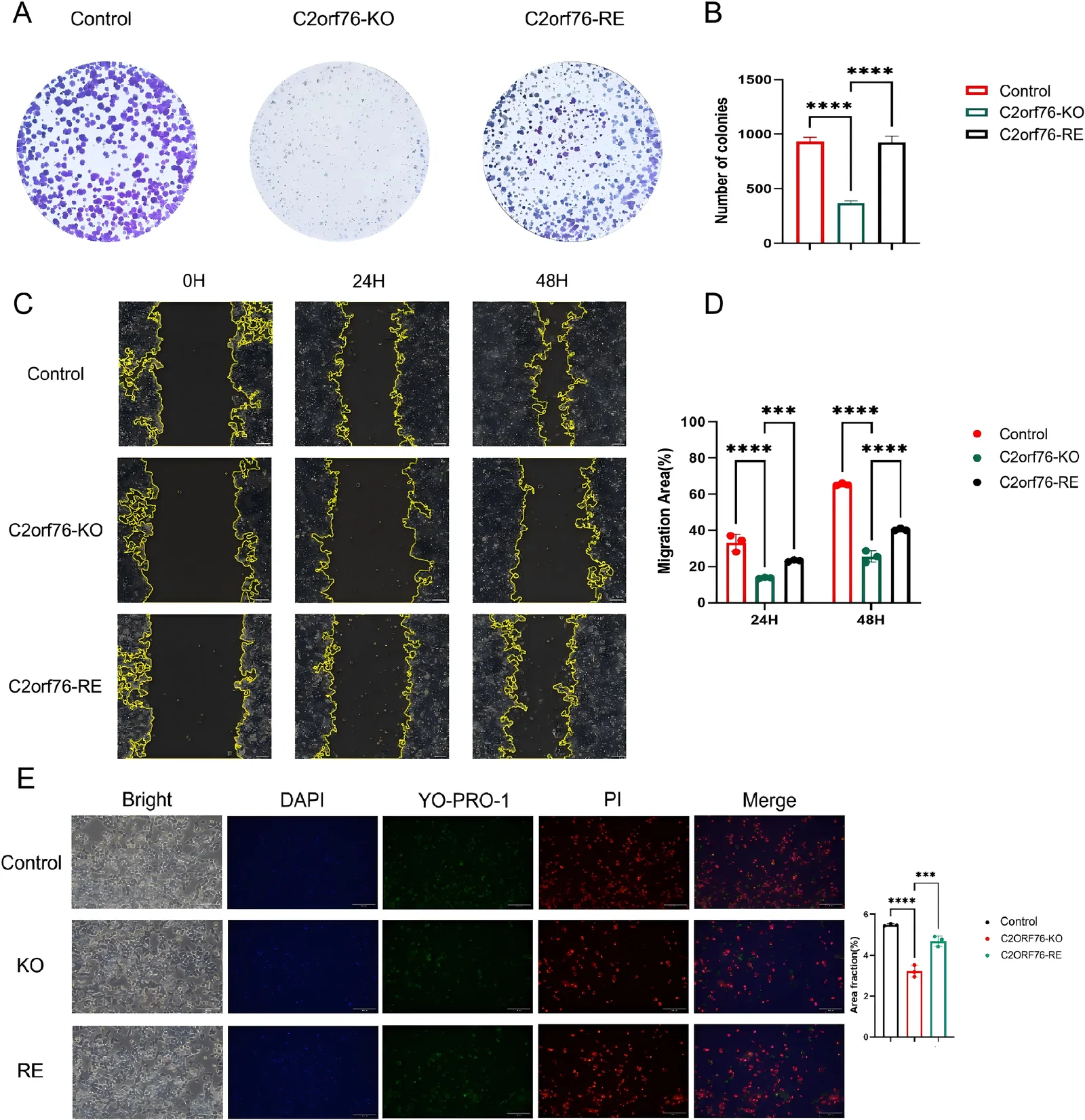
Rescue of C2orf76 expression reverses knockout-induced phenotypes in CRC cells. **(A, B)** Representative images **(A)** and quantitative analysis **(B)** of colony formation assays. KO cells showed reduced colony formation, which was restored in RE cells. **(C, D)** Representative images **(C)** and quantitative analysis **(D)** of wound healing assays. The impaired migratory capacity observed in KO cells was significantly rescued in RE cells. Scale bar, 50 μm. **(E)** Fluorescence staining using YO-PRO-1 (green) and propidium iodide (PI, red) in cisplatin-treated cells. KO cells exhibited reduced positive staining, indicating chemoresistance, which was reversed upon C2orf76 re-expression in RE cells. Scale bar, 100 μm. *p < 0.05, **p < 0.01, ***p < 0.001, ****p < 0.0001; NS, not significant.

To verify that the phenotypes observed upon C2orf76 knockout were specifically attributable to C2orf76 deficiency rather than off-target effects, we generated rescue (RE) HCT116 cells by re-expressing C2orf76 in the knockout background. Colony formation assays revealed that the reduced proliferative capacity of KO cells was markedly restored upon C2orf76 re-expression in RE cells (**Figures 10A, B**). Wound healing assays further demonstrated that the impaired migratory capacity induced by C2orf76 knockout was significantly attenuated in RE cells (**Figures 10C, D**). Notably, fluorescence staining using YO-PRO-1 and propidium iodide (PI) revealed that the cisplatin-resistant phenotype observed in KO cells was reversed by C2orf76 re-expression (**Figure 10E**). Collectively, these rescue experiments indicate that the phenotypic alterations induced by C2orf76 knockout are specifically mediated by C2orf76 deficiency.

**Figure 11:**
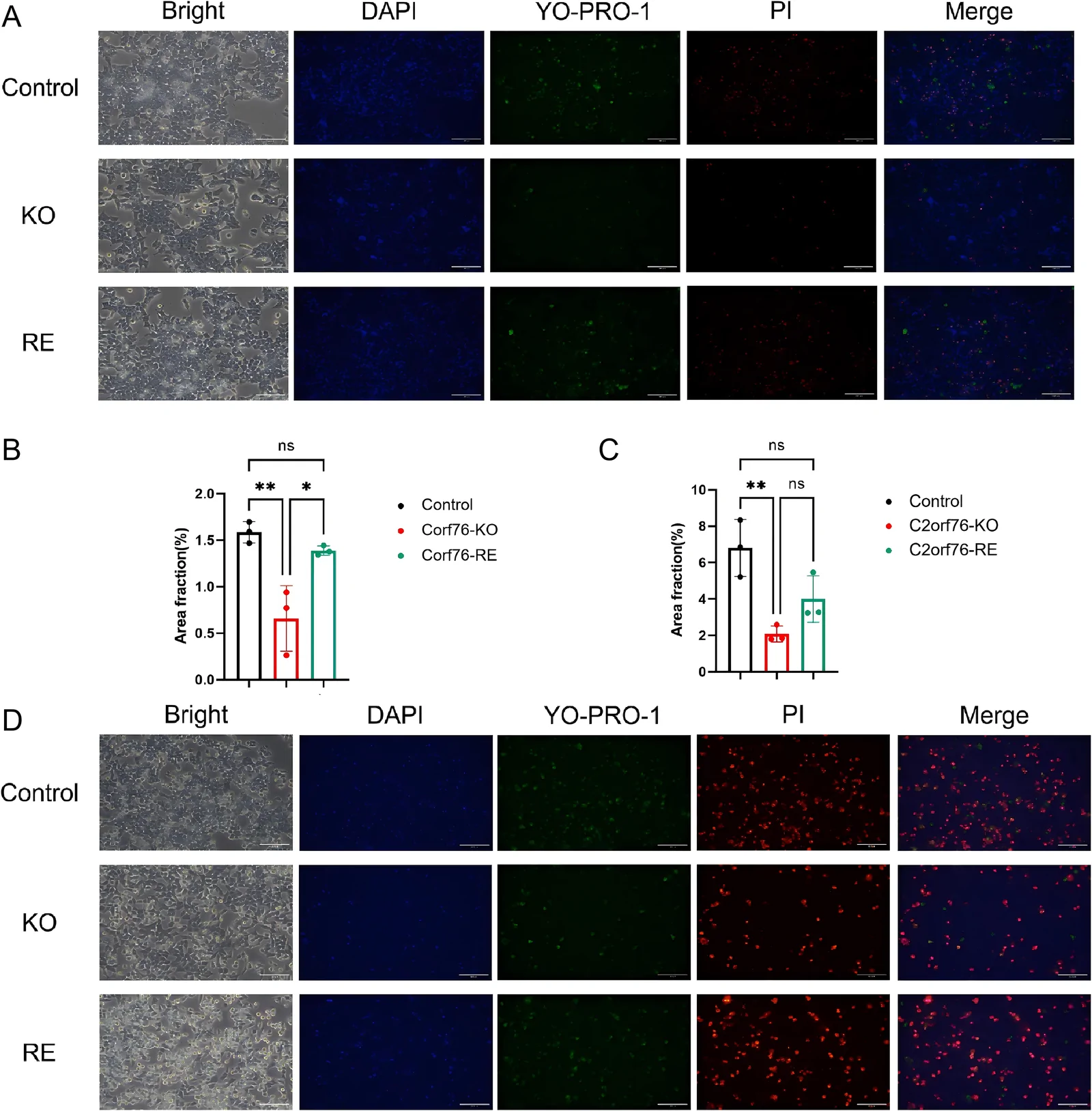
C2orf76 knockout enhances resistance of CRC cells to irinotecan and oxaliplatin, which is reversed by C2orf76 re-expression. **(A)** Representative fluorescence images of cells treated with irinotecan, stained with DAPI (blue, nuclei), YO-PRO-1 (green, early apoptotic cells), and propidium iodide (PI, red, late apoptotic/necrotic cells). Scale bar, 100 μm. **(B)** Quantification of cell death (percentage of YO-PRO-1^+^/PI^+^ cells) from three independent experiments for irinotecan treatment (n = 3). **(C)** Quantification of cell death for oxaliplatin treatment. **(D)** Representative fluorescence images of cells treated with oxaliplatin, stained as in **(A)**. *p < 0.05, **p < 0.01, ***p < 0.001, ****p < 0.0001; NS, not significant.

**Figure 12:**
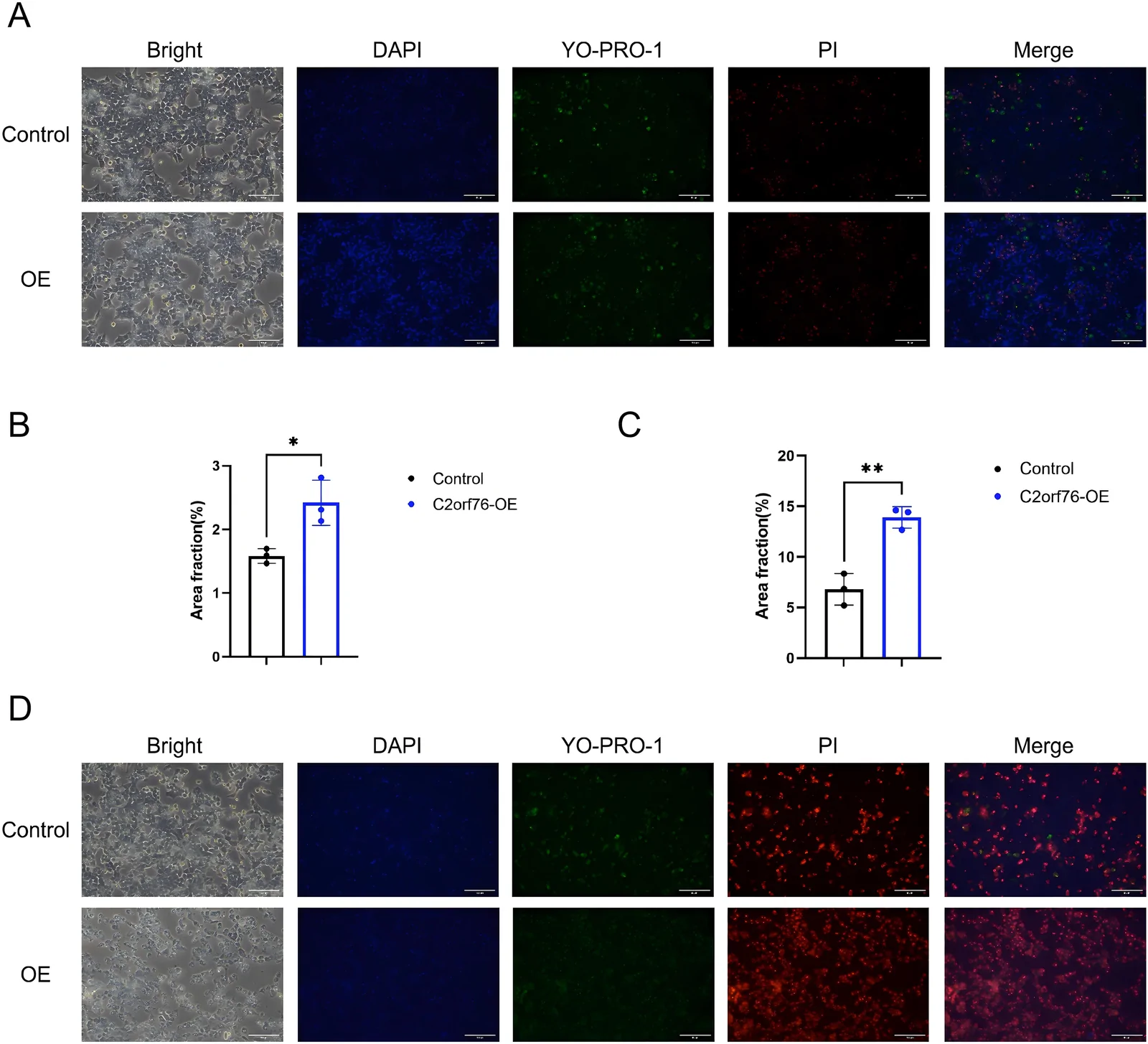
C2orf76 overexpression enhances chemosensitivity to irinotecan and oxaliplatin. **(A)** Representative fluorescence images of cells treated with irinotecan, stained with DAPI (blue), YO-PRO-1 (green), and PI (red). OE cells show increased positive staining compared with control. Scale bar, 100 μm. **(B)** Quantification of cell death after irinotecan treatment. **(C)** Quantification of cell death after oxaliplatin treatment. **(D)** Representative fluorescence images of cells treated with oxaliplatin, stained as in **(A)**. *p < 0.05, **p < 0.01, ***p < 0.001, ****p < 0.0001; NS, not significant.

To validate the role of C2orf76 knockout in chemoresistance of colorectal cancer (CRC), we treated cells with two additional clinically used chemotherapeutic agents, irinotecan and oxaliplatin. Fluorescence staining with DAPI (nuclei), YO-PRO-1, and propidium iodide was performed to assess cell death. As shown in **Figures 11A–D**, C2orf76-deficient cells exhibited a significantly lower percentage of YO-PRO-1^+^/PI^+^ positive cells compared with control cells upon either irinotecan or oxaliplatin treatment, indicating reduced cell death and thus enhanced drug resistance. Importantly, re-expression of C2orf76 in the knockout cells reversed this resistance phenotype. To further examine whether increased C2orf76 expression sensitizes CRC cells to chemotherapy, we treated C2orf76 (OE) cells with irinotecan or oxaliplatin. As shown in **Figures 12A–D**, upon irinotecan or oxaliplatin treatment, OE cells displayed a significantly higher percentage of YO-PRO-1^+^/PI^+^ cells compared with control cells, demonstrating that C2orf76 overexpression promotes chemosensitivity to both drugs. Collectively, these results support a role for C2orf76 as a negative regulator of chemotherapy response in CRC, where its loss confers resistance and its gain sensitizes cells to treatment.

### 3.6 Effects of C2orf76 regulation on chromosomal instability in colorectal cancer

**Figure 13:**
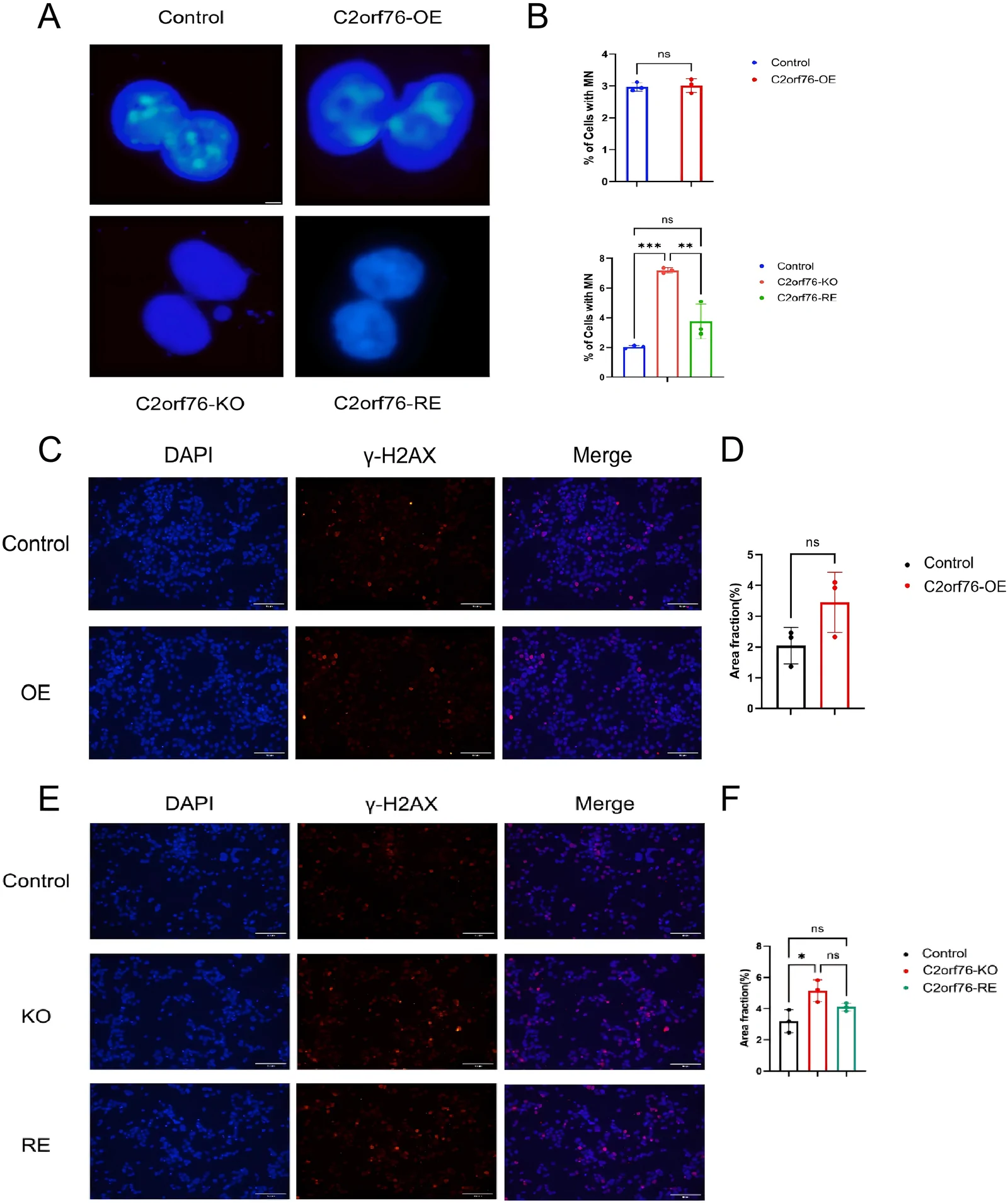
C2orf76 knockout disrupt chromosomal stability. **(A, B)** CBMN assay for Micronucleus (MN) formation (DAPI-stained nuclei). MN frequency (%) was scored in binucleated cells (≥1000 per group). KO had a significant increase in MN frequency; OE and RE were similar to control. Scale bar, 5 μm. **(C-F)** γ-H2AX immunofluorescence for DNA double-strand breaks. KO cells exhibited significantly more foci than controls, while OE and RE cells showed comparable levels. Scale bar, 100 μm. *p < 0.05, **p < 0.01, ***p < 0.001, ****p < 0.0001; NS, not significant.

The effect of C2orf76 on chromosomal stability in colorectal cancer cells was evaluated using the cytokinesis-block micronucleus (CBMN) assay and γ-H2AX immunofluorescence staining. Knockdown of C2orf76 significantly increased the frequency of micronucleus formation, and this effect was abolished by rescue expression of C2orf76. C2orf76-overexpressing cells showed micronucleus frequencies comparable to those of the control group (**Figures 13A, B**; **Supplementary Figure 7**). In parallel, upon cisplatin treatment, γ‑H2AX foci, a sensitive marker of DNA, were markedly elevated in C2orf76-knockdown cells relative to controls, whereas both rescue and overexpression groups exhibited foci numbers similar to those of the control (**Figures 13C–F**). Collectively, these findings demonstrate that C2orf76 depletion compromises chromosomal stability, as evidenced by increased micronuclei and DNA damage foci. Furthermore, these results are consistent with the GO and KEGG pathway analyses derived from our RNA-sequencing data and qPCR results.

**Figure 14:**
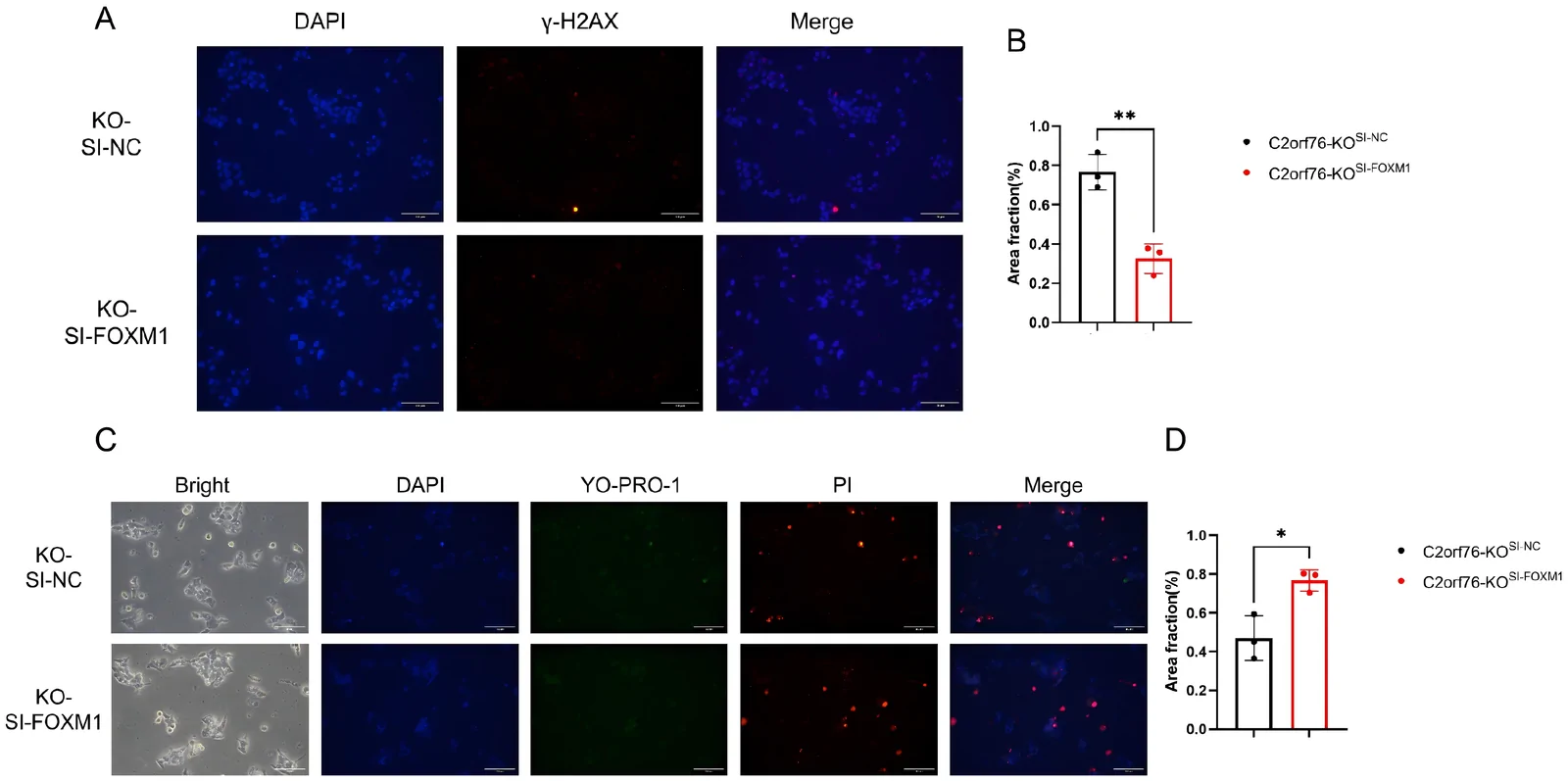
FOXM1 knockdown resensitizes C2orf76-knockout CRC cells to cisplatin and reduces γ-H2AX foci. **(A)** Representative fluorescence images of γ-H2AX foci (red) and nuclei (DAPI, blue) in KO cells transfected with siNC or siFOXM1. Scale bar, 100 μm. **(B)** Quantification of γ-H2AX foci from three independent experiments. FOXM1 knockdown significantly decreased foci formation, indicating reduced DNA double-strand break accumulation. **(C)** Representative fluorescence images of C2orf76-knockout **(KO)** HCT116 cells transfected with control siRNA (siNC) or FOXM1 siRNA (siFOXM1), followed by cisplatin treatment. Cells were stained with YO-PRO-1 (green) and propidium iodide (PI, red). Scale bar, 100 μm. **(D)** Quantification of cell death (percentage of YO-PRO-1^+^/PI^+^ cells) from three independent experiments. FOXM1 knockdown significantly increased cisplatin-induced cell death in KO cells compared with siNC-transfected KO cells. *p < 0.05, **p < 0.01, ***p < 0.001, ****p < 0.0001; NS, not significant.

To determine whether FOXM1 mediates C2orf76-knockout-induced chemoresistance and chromosomal instability, we performed siRNA-mediated knockdown of FOXM1 in C2orf76-knockout (KO) HCT116 cells, followed by cisplatin treatment. Fluorescence staining with YO-PRO-1 and propidium iodide (PI) revealed that FOXM1 knockdown significantly increased the percentage of dead cells (YO-PRO-1^+^/PI^+^) in KO cells upon cisplatin challenge, compared with KO cells transfected with control siRNA (**Figures 14C, D**). Moreover, γ-H2AX foci formation, was markedly reduced in FOXM1-depleted KO cells, indicating alleviated DNA damage and repair defects (**Figures 14A, B**). Collectively, these results suggest that FOXM1 functions as a downstream effector of C2orf76 in modulating chemoresistance and genomic instability in CRC cells.

### 3.7 Effects of C2orf76 modulation on general tumor phenotypes in SW480 cells

**Figure 15:**
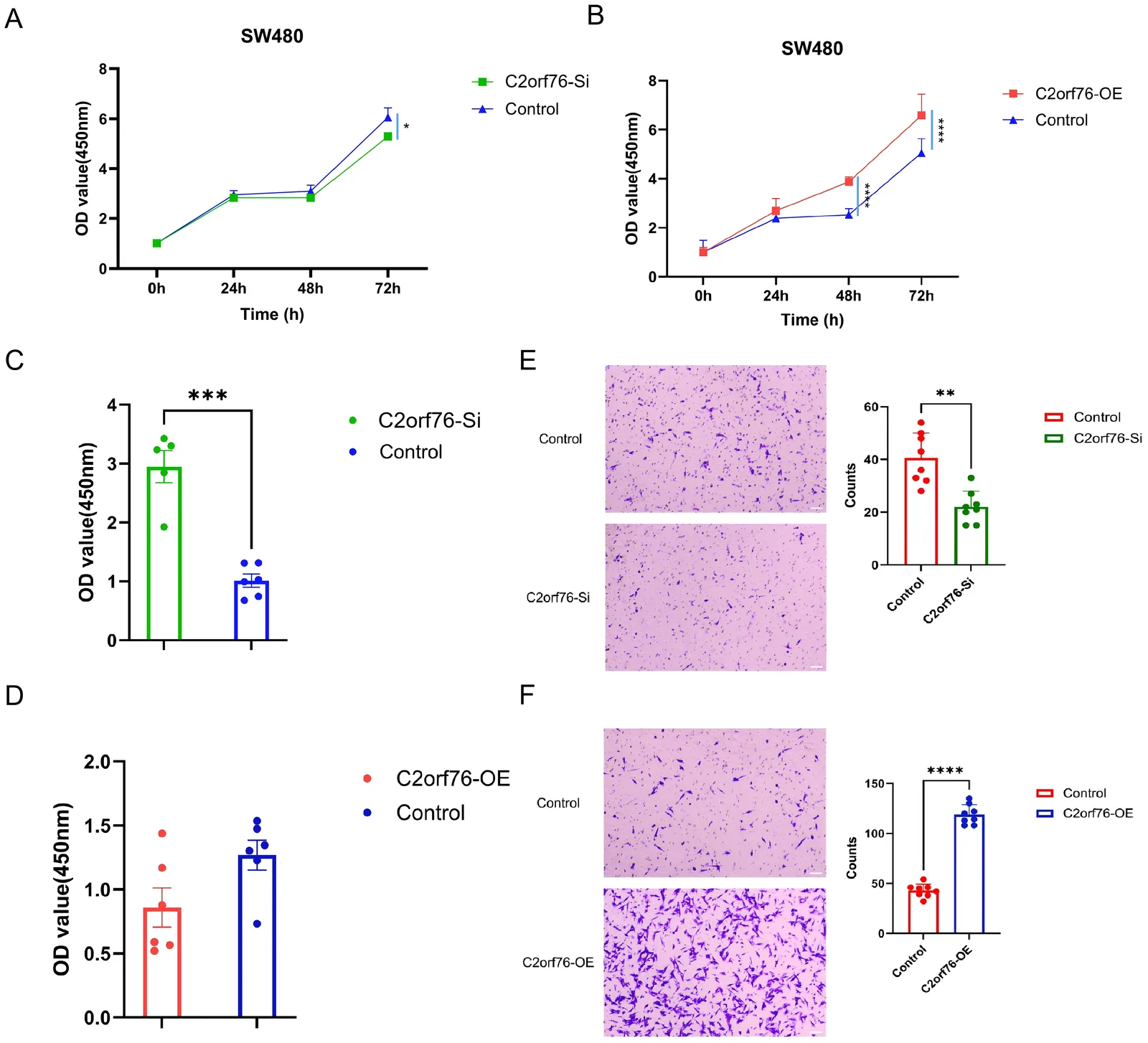
C2orf76 differentially regulates proliferation, migration, and chemoresistance in SW480 cells. **(A)** Proliferation curves of control and C2orf76‑knockdown(Si) SW480 cells measured by CCK-8 over time. A significant reduction in cell growth was observed only at 72 h (n = 3). **(B)** Proliferation curves of control and C2orf76-overexpressing (OE) SW480 cells, showing that OE significantly enhances proliferation compared with control. **(C)** Quantification of cell viability after cisplatin treatment in control and C2orf76‑knockdown cells. C2orf76‑knockdown cells display significantly lower cell death, indicating increased chemoresistance. **(D)** Quantification of cell viability after cisplatin treatment in control and OE cells. No significant difference is observed. **(E, F)** Representative images and Quantitative analysis of cell migration for control, C2orf76‑knockdown, and OE groups. Scale bar, 100 μm. C2orf76‑knockdown significantly inhibits migration, whereas OE promotes migration. *p < 0.05, **p < 0.01, ***p < 0.001, ****p < 0.0001; NS, not significant.

To further validate the role of C2orf76 in colorectal cancer, we examined the effects of modulating C2orf76 expression on proliferation, migration, and chemoresistance in SW480 cells, a colorectal cancer cell line with greater chromosomal instability compared to HCT116 cells. In terms of cell proliferation, the growth-inhibitory effect of C2orf76 knockdown in SW480 cells was weaker and occurred later than that in HCT116 cells, with a significant difference only appearing at 72 hours (**Figure 15A**). Moreover, unlike in HCT116 cells, overexpression of C2orf76 significantly promoted proliferation in SW480 cells (**Figure 15B**). Regarding cancer cell migration, the two cell lines showed consistent results: C2orf76 knockdown inhibited migration, while C2orf76 overexpression promoted migration (**Figures 15E, F**). Furthermore, in SW480 cells, C2orf76 knockdown also enhanced chemoresistance to cisplatin treatment, but overexpression of C2orf76 did not significantly alter chemosensitivity (**Figures 15C, D**).

## 4 Discussion

Research indicates that CRC with a high level of CIN is immunosuppressive and resistant to both immune checkpoint blockade and conventional chemotherapy (23). In this study, the CIN-associated gene C2orf76 was highly expressed in CRC cells and showed potential correlations with immunotherapy-related signatures and immune infiltration based on bioinformatics analyses. High C2orf76 expression predicted better prognosis, especially in TP53-mutant patients. Interestingly, while C2orf76 knockout reduced cell viability and metastasis, it concurrently enhanced chemoresistance and pyroptosis resistance, suggesting context-dependent and pleiotropic functions. RNA-seq linked C2orf76 to pathways governing chromosomal stability, cell cycle, platinum resistance, and p53 signaling; correlations with CDT1, FOXM1, and other CIN-associated genes were validated by RT-qPCR. The CBMN assay and γ-H2AX staining confirmed its role in chromosomal stability. Notably, the resistance phenotypes induced by C2orf76 ablation were reversed by re-expression, while overexpression promoted migration and chemosensitivity. Consistent results in SW480 cells ensured generalizability. Collectively, these findings position C2orf76 as a multifaceted regulator bridging chromosomal stability, drug response, and tumor aggressiveness, warranting further exploration as a potential therapeutic target or biomarker.

The phenotypic discrepancy between patient survival data and the C2orf76 knockout phenotypes suggests an atypical role for C2orf76 in CRC. Notably, TP53 inhibition appeared to restore (rather than suppress) the viability of C2orf76-deficient cells, hinting at potential crosstalk between these two pathways, although this remains to be experimentally validated. As a well-known tumor suppressor (24), P53 is frequently mutated in CRC, with TP53 mutations occurring in approximately 43% of cases (25, 26). Under TP53-mutant conditions, the growth-suppressive effect of low C2orf76 expression may be overshadowed by the dominant pro-resistance function of C2orf76 (27), which may partly explain the worse prognosis observed in these patients. RNA-seq analysis also linked C2orf76 to the p53 signaling pathway, but the functional relevance of this association warrants further investigation.

RNA-seq of C2orf76-knockout CRC cells revealed enrichment in pathways governing cell cycle, chromosome segregation, and DNA mismatch repair. RT-qPCR validated the dysregulation of several CIN-associated genes, including CDT1 and FOXM1. While FOXM1 protein levels were elevated in line with its mRNA, CDT1 protein paradoxically decreased despite transcriptional upregulation. Chromatin licensing and DNA replication factor 1 (CDT1) is essential for pre-replicative complex assembly and DNA replication licensing (28), and its overexpression in CRC mice drives DNA damage response activation, micronuclei formation, and chromosome segregation errors (29). Given that C2orf76 ablation itself induces CIN, transcriptional changes of these genes may reflect a cellular stress response. This interpretation is further supported by the concurrent downregulation of other CIN-associated genes detected by qPCR. Fork-head Box M1 (FOXM1) plays dual roles in genomic instability: its normal function preserves stability (30, 31), whereas its dysregulation—whether loss or overexpression—promotes chromosomal abnormalities and DNA damage (32–35). Notably, FOXM1 upregulation confers cisplatin resistance in breast cancer (36). Therefore, the sustained FOXM1 elevation, in contrast to the downregulation of other CIN-related genes, may be primarily driven by its chemoresistance-promoting function. In subsequent experiments, FOXM1 depletion in C2orf76-knockout cells rescued chemosensitivity and chromosomal stability, implicating FOXM1 as a downstream mediator of C2orf76 in these processes. Collectively, these layered regulatory adjustments likely contribute to the survival advantage and attenuated drug response observed in C2orf76-deficient cells.

Chromosomal instability is a ubiquitous feature of human tumors, affecting more than 70% of colorectal cancer cases (37). Although aneuploidy is prevalent in tumors, its experimental induction generally impairs cellular proliferation (38, 39), a finding consistent with the growth-suppressive effect of C2orf76 knockout in our study. Conversely, CIN-conferred phenotypic plasticity enables cancer cells to adapt to stress, frequently reducing therapeutic sensitivity and correlating with poor clinical outcomes (40–42). This aligns with our observation that C2orf76 ablation confers chemoresistance in CRC cells. As a critical regulator of DNA repair, TP53—when mutated—compromises genomic integrity and promotes extensive chromosomal abnormalities (43), a common event in CRC (44). Notably, TP53 mutation may interact with C2orf76 to drive chromosomal instability, potentially accounting for the survival disparities observed between patients with low versus high C2orf76 expression.

To further validate the role of C2orf76 in CIN-driven CRC, we employed SW480 cells, a colorectal cancer cell line that harbors endogenous TP53 mutation and exhibits higher chromosomal instability. In these cells, the effects of C2orf76 modulation on migration were consistent with those observed in HCT116 cells. Regarding proliferation, we observed a similar rescue of the growth inhibition induced by C2orf76 knockdown, which parallels the TP53-siRNA findings in C2orf76-KO HCT116 cells. Moreover, C2orf76 overexpression significantly promoted proliferation in SW480 cells. Notably, C2orf76 ablation also enhanced chemoresistance in SW480 cells, suggesting that its pro-resistance function may extend beyond CIN induction and involve additional mechanisms, such as the modulation of pyroptosis.

Pyroptosis, a pro-inflammatory form of programmed cell death, improves the response to immunotherapy (6). Even pyroptosis of a small proportion of tumor cells is sufficient to remodel the tumor immune microenvironment and subsequently activate a potent T-cell-mediated anti-tumor immune response (45). In our study, C2orf76 knockout reduced GSDME cleavage—a key pyroptosis executor—in CRC cells, and modulation of C2orf76 altered cisplatin-induced cell death, implicating its involvement in pyroptosis regulation. FOXM1 has been reported to alleviate podocyte pyroptosis in diabetic nephropathy (46) and to bind the PD-L1 promoter, enhancing its expression in NSCLC (47). Given that FOXM1 is upregulated upon C2orf76 ablation and functions as a downstream mediator in our system, it is plausible that FOXM1 contributes, at least in part, to the pyroptosis‑resistant phenotype we observed—consistent with its reported anti‑pyroptotic function. Whether FOXM1 also modulates immune‑related pathways in CRC under these conditions remains an open question that merits further investigation.

Tumor immunity adds another layer of complexity to CIN-driven tumorigenesis. Colorectal cancers with high CIN are often immunosuppressive and resistant to immune checkpoint blockade therapy (13, 17, 48), although increased neoantigen load in some CIN-high tumors may potentially enhance immunotherapy responses (49–51). The MSI and CIN-high phenotypes are largely mutually exclusive, as combined genomic instability would likely be intolerable for cell survival (52, 53). MSI cancers typically respond better to immunotherapy, whereas CIN tumors frequently exhibit immune evasion and poor outcomes (54–56). Importantly, the majority of tumors develop CIN and remain refractory to current immunotherapies (57); enhancing their sensitivity is therefore a major research focus (58, 59). Prior studies have shown that modulating individual genes involved in genomic instability can alter immunotherapy responses (23, 60), providing a theoretical basis for exploring C2orf76 in this context. In our study, the potential correlations between C2orf76 and immune-related signatures were primarily derived from bioinformatics analyses and lack experimental validation. Moreover, our findings are limited by the absence of *in vivo* animal models and clinical sample verification. Given the dual roles of C2orf76 in chromosomal stability and pyroptosis, its precise targeting may hold translational potential, but whether this translates into enhanced immunotherapy efficacy in CRC requires further investigation.

## 5 Conclusions

Through screening of online datasets and bioinformatic analysis, we identified C2orf76 as a gene associated with genomic instability, with potential correlations to immunotherapy and immune cell infiltration. By constructing C2orf76-knockout, rescue, and overexpression colorectal cancer cell lines, we found that C2orf76 ablation significantly reduced tumor cell viability and migration, while concurrently increasing chromosomal instability and conferring resistance to chemotherapy-induced cell death and pyroptosis. Importantly, these resistance phenotypes were effectively reversed by C2orf76 re-expression in rescue experiments. Conversely, C2orf76 overexpression promoted cell migration and enhanced sensitivity to cisplatin. Transcriptome sequencing revealed that C2orf76 knockout-induced changes were enriched in pathways governing chromosomal regulation, cell cycle, platinum resistance, p53 signaling, and other cancer-related processes. Given the established links between chromosomal instability and CRC phenotypic traits, together with the observed effects of C2orf76 on pyroptosis and the validation by rescue experiments, this study suggests C2orf76 as a potential target for modulating CIN-driven therapy resistance. Nonetheless, this work offers a new genetic basis for CRC prognosis and warrants further exploration of C2orf76 as a candidate for therapeutic intervention.

## Data Availability

The datasets analyzed in this study are publicly available. GEO datasets GSE30540 and GSE-34489 can be accessed via the NCBI GEO database (https://www.ncbi.nlm.nih.gov/geo/). TCGA data were accessed and analyzed using the cBioPortal platform (https://www.cbioportal.org/). The original contributions presented in the study are included in the article/**Supplementary Material**. Further inquiries can be directed to the corresponding authors.

## References

[1] Akimoto N Ugai T Zhong R Hamada T Fujiyoshi K Giannakis M . Rising incidence of early-onset colorectal cancer - a call to action. Nat Rev Clin Oncol. (2021) 18:230–43. doi: 10.1038/s41571-020-00445-1 33219329 PMC7994182

[2] Heinimann K . Hereditary colorectal cancer: clinics, diagnostics and management. Ther Umsch. (2018) 75:601–6. doi: 10.1024/0040-5930/a001046 31232663

[3] Biller LH Schrag D . Diagnosis and treatment of metastatic colorectal cancer: a review. Jama. (2021) 325:669–85. doi: 10.1001/jama.2021.0106 33591350

[4] Sahin IH Ciombor KK Diaz LA Yu J Kim R . Immunotherapy for microsatellite stable colorectal cancers: challenges and novel therapeutic avenues. Am Soc Clin Oncol Educ Book Am Soc Clin Oncol Annu Meeting. (2022) 42:1–12. doi: 10.1200/edbk_349811 35658496

[5] Xiao Q Nobre A Piñeiro P Berciano-Guerrero M Alba E Cobo M . Genetic and epigenetic biomarkers of immune checkpoint blockade response. J Clin Med. (2020) 9(1). doi: 10.3390/jcm9010286 31968651 PMC7019273

[6] Li L Zhao L Zhou D Yu Y Zhang P Zheng J . Targeting pyroptosis reverses Kiaa1199-mediated immunotherapy resistance in colorectal cancer. J Immunother Cancer. (2025) 13(2). doi: 10.1136/jitc-2024-010000 40010767 PMC11865760

[7] Watanabe T Kobunai T Yamamoto Y Matsuda K Ishihara S Nozawa K . Chromosomal instability (Cin) phenotype, Cin high or Cin low, predicts survival for colorectal cancer. J Clin Oncol. (2012) 30:2256–64. doi: 10.1200/jco.2011.38.6490 22547595

[8] Xu J Qin S Yi Y Gao H Liu X Ma F . Delving into the heterogeneity of different breast cancer subtypes and the prognostic models utilizing scrna-seq and bulk rna-seq. Int J Mol Sci. (2022) 23(17). doi: 10.3390/ijms23179936 36077333 PMC9456551

[9] Dybska E Nowak JK Walkowiak J . Transcriptomic context of Runx3 expression in monocytes: a cross-sectional analysis. Biomedicines. (2023) 11(6). doi: 10.3390/biomedicines11061698 37371794 PMC10296263

[10] Paliwal A Temkin AM Kerkel K Yale A Yotova I Drost N . Comparative anatomy of chromosomal domains with imprinted and non-imprinted allele-specific DNA methylation. PloS Genet. (2013) 9:e1003622. doi: 10.1371/journal.pgen.1003622 24009515 PMC3757050

[11] Ippolito MR Martis V Martin S Tijhuis AE Hong C Wardenaar R . Gene copy-number changes and chromosomal instability induced by aneuploidy confer resistance to chemotherapy. Dev Cell. (2021) 56:2440–2454.e6. doi: 10.1016/j.devcel.2021.07.006 34352223

[12] Swanton C Nicke B Schuett M Eklund AC Ng C Li Q . Chromosomal instability determines taxane response. Proc Natl Acad Sci USA. (2009) 106:8671–6. doi: 10.1073/pnas.0811835106 19458043 PMC2688979

[13] Davoli T Uno H Wooten EC Elledge SJ . Tumor aneuploidy correlates with markers of immune evasion and with reduced response to immunotherapy. Science. (2017) 355(6322). doi: 10.1126/science.aaf8399 28104840 PMC5592794

[14] Bakhoum SF Ngo B Laughney AM Cavallo JA Murphy CJ Ly P . Chromosomal instability drives metastasis through a cytosolic DNA response. Nature. (2018) 553:467–72. doi: 10.1038/nature25432 29342134 PMC5785464

[15] Carter SL Eklund AC Kohane IS Harris LN Szallasi Z . A signature of chromosomal instability inferred from gene expression profiles predicts clinical outcome in multiple human cancers. Nat Genet. (2006) 38:1043–8. doi: 10.1038/ng1861 16921376

[16] Crockford A Zalmas LP Grönroos E Dewhurst SM McGranahan N Cuomo ME . Cyclin D mediates tolerance of genome-doubling in cancers with functional P53. Ann Oncol Off J Eur Soc For Med Oncol. (2017) 28:149–56. doi: 10.1093/annonc/mdw612 28177473 PMC5391719

[17] Bakhoum SF Cantley LC . The multifaceted role of chromosomal instability in cancer and its microenvironment. Cell. (2018) 174:1347–60. doi: 10.1016/j.cell.2018.08.027 30193109 PMC6136429

[18] Hong C Schubert M Tijhuis AE Requesens M Roorda M van den Brink A . Cgas-Sting drives the Il-6-dependent survival of chromosomally instable cancers. Nature. (2022) 607:366–73. doi: 10.1038/s41586-022-04847-2 35705809

[19] Dai D Chen C Lu C Guo Y Li Q Sun C . Apoptosis, autophagy, ferroptosis, and pyroptosis in cisplatin-induced ototoxicity and protective agents. Front Pharmacol. (2024) 15:1430469. doi: 10.3389/fphar.2024.1430469 39380912 PMC11459463

[20] Yan H Luo B Wu X Guan F Yu X Zhao L . Cisplatin induces pyroptosis via activation of Meg3/Nlrp3/Caspase-1/Gsdmd pathway in triple-negative breast cancer. Int J Biol Sci. (2021) 17:2606–21. doi: 10.7150/ijbs.60292 34326697 PMC8315016

[21] Gao W Wang X Zhou Y Wang X Yu Y . Autophagy, ferroptosis, pyroptosis, and necroptosis in tumor immunotherapy. Signal Transduct Target Ther. (2022) 7:196. doi: 10.1038/s41392-022-01046-3 35725836 PMC9208265

[22] Man SM Karki R Kanneganti TD . Molecular mechanisms and functions of pyroptosis, inflammatory caspases and inflammasomes in infectious diseases. Immunol Rev. (2017) 277:61–75. doi: 10.1111/imr.12534 28462526 PMC5416822

[23] Liu G Zhang Y Cao Z Zhao Z . Targeting Kif18a triggers antitumor immunity and enhances efficiency of Pd-1 blockade in colorectal cancer with chromosomal instability phenotype. Cell Death Discov. (2025) 11:130. doi: 10.1038/s41420-025-02437-5 40175357 PMC11965295

[24] Whibley C Pharoah PD Hollstein M . P53 polymorphisms: cancer implications. Nat Rev Cancer. (2009) 9:95–107. doi: 10.1038/nrc2584 19165225

[25] Sabapathy K Lane DP . Therapeutic targeting of P53: all mutants are equal, but some mutants are more equal than others. Nat Rev Clin Oncol. (2018) 15:13–30. doi: 10.1038/nrclinonc.2017.151 28948977

[26] Li W Li L Yang H Shi C Lei Z Guo L . Unraveling the role of Tp53 in colorectal cancer therapy: from wild-type regulation to mutant. Front Biosci (Landmark Ed). (2024) 29:272. doi: 10.31083/j.fbl2907272 39082342

[27] Li H Rokavec M Jiang L Horst D Hermeking H . Antagonistic effects of P53 and Hif1a on Microrna-34a regulation of Ppp1r11 and Stat3 and hypoxia-induced epithelial to mesenchymal transition in colorectal cancer cells. Gastroenterology. (2017) 153(10):505–20. doi: 10.1053/j.gastro.2017.04.017 28435028

[28] Zhang H . Regulation of DNA replication licensing and re-replication by Cdt1. Int J Mol Sci. (2021) 22. doi: 10.3390/ijms22105195 34068957 PMC8155957

[29] Petropoulos M Champeris Tsaniras S Nikou S Maxouri S Dionellis VS Kalogeropoulou A . Cdt1 overexpression drives colorectal carcinogenesis through origin overlicensing and DNA damage. J Pathol. (2023) 259:10–20. doi: 10.1002/path.6017 36210634

[30] Barger CJ Branick C Chee L Karpf AR . Pan-cancer analyses reveal genomic features of Foxm1 overexpression in cancer. Cancers (Basel). (2019) 11(2). doi: 10.3390/cancers11020251 30795624 PMC6406812

[31] Laoukili J Kooistra MR Brás A Kauw J Kerkhoven RM Morrison A . Foxm1 is required for execution of the mitotic programme and chromosome stability. Nat Cell Biol. (2005) 7:126–36. doi: 10.1038/ncb1217 15654331

[32] Pan F Chocarro S Ramos M Chen Y Alonso de la Vega A Somogyi K . Foxm1 is critical for the fitness recovery of chromosomally unstable cells. Cell Death Dis. (2023) 14:430. doi: 10.1038/s41419-023-05946-2 37452072 PMC10349069

[33] Teh MT Gemenetzidis E Chaplin T Young BD Philpott MP . Upregulation of Foxm1 induces genomic instability in human epidermal keratinocytes. Mol Cancer. (2010) 9:45. doi: 10.1186/1476-4598-9-45 20187950 PMC2907729

[34] Weiler SME Pinna F Wolf T Lutz T Geldiyev A Sticht C . Induction of chromosome instability by activation of Yes-associated protein and Forkhead box M1 in liver cancer. Gastroenterology. (2017) 152:2037–2051.e22. doi: 10.1053/j.gastro.2017.02.018 28249813

[35] Jeffery JM Kalimutho M Johansson P Cardenas DG Kumar R Khanna KK . Fbxo31 protects against genomic instability by capping Foxm1 levels at the G2/M transition. Oncogene. (2017) 36:1012–22. doi: 10.1038/onc.2016.268 27568981

[36] Kwok JM Peck B Monteiro LJ Schwenen HD Millour J Coombes RC . Foxm1 confers acquired cisplatin resistance in breast cancer cells. Mol Cancer Res. (2010) 8:24–34. doi: 10.1158/1541-7786.Mcr-09-0432 20068070 PMC2809047

[37] Papaccio F Cabeza-Segura M García-Micó B Gimeno-Valiente F Zúñiga-Trejos S Gambardella V . Decoding chromosomal instability insights in Crc by integrating omics and patient-derived organoids. J Exp Clin Cancer Res. (2025) 44:77. doi: 10.1186/s13046-025-03308-8 40022181 PMC11869439

[38] Sheltzer JM Amon A . The aneuploidy paradox: costs and benefits of an incorrect karyotype. Trends Genet. (2011) 27:446–53. doi: 10.1016/j.tig.2011.07.003 21872963 PMC3197822

[39] Sheltzer JM Ko JH Replogle JM Habibe Burgos NC Chung ES Meehl CM . Single-chromosome gains commonly function as tumor suppressors. Cancer Cell. (2017) 31:240–55. doi: 10.1016/j.ccell.2016.12.004 28089890 PMC5713901

[40] Lukow DA Sausville EL Suri P Chunduri NK Wieland A Leu J . Chromosomal instability accelerates the evolution of resistance to anti-cancer therapies. Dev Cell. (2021) 56:2427–2439.e4. doi: 10.1016/j.devcel.2021.07.009 34352222 PMC8933054

[41] Lukow DA Sheltzer JM . Chromosomal instability and aneuploidy as causes of cancer drug resistance. Trends Cancer. (2022) 8:43–53. doi: 10.1016/j.trecan.2021.09.002 34593353

[42] Smeets D Miller IS O'Connor DP Das S Moran B Boeckx B . Copy number load predicts outcome of metastatic colorectal cancer patients receiving bevacizumab combination therapy. Nat Commun. (2018) 9:4112. doi: 10.1038/s41467-018-06567-6 30291241 PMC6173768

[43] Janic A Abad E Amelio I . Decoding P53 tumor suppression: a crosstalk between genomic stability and epigenetic control? Cell Death Differ. (2025) 32:1–8. doi: 10.1038/s41418-024-01259-9 38379088 PMC11742645

[44] Michel M Kaps L Maderer A Galle PR Moehler M . The role of P53 dysfunction in colorectal cancer and its implication for therapy. Cancers (Basel). (2021) 13(10). doi: 10.3390/cancers13102296 34064974 PMC8150459

[45] Zhou Z He H Wang K Shi X Wang Y Su Y . Granzyme a from cytotoxic lymphocytes cleaves Gsdmb to trigger pyroptosis in target cells. Science. (2020) 368(6484). doi: 10.1126/science.aaz7548 32299851

[46] Xu X Zhang L Hua F Zhang C Zhang C Mi X . Foxm1-activated Sirt4 inhibits Nf-Kb signaling and Nlrp3 inflammasome to alleviate kidney injury and podocyte pyroptosis in diabetic nephropathy. Exp Cell Res. (2021) 408:112863. doi: 10.1016/j.yexcr.2021.112863 34626587

[47] Madhi H Lee JS Choi YE Li Y Kim MH Choi Y . Foxm1 inhibition enhances the therapeutic outcome of lung cancer immunotherapy by modulating Pd-L1 expression and cell proliferation. Advanced Sci (Weinheim Baden-Wurttemberg Germany). (2022) 9:e2202702. doi: 10.1002/advs.202202702 35975458 PMC9561767

[48] Heiser CN Simmons AJ Revetta F McKinley ET Ramirez-Solano MA Wang J . Molecular cartography uncovers evolutionary and microenvironmental dynamics in sporadic colorectal tumors. Cell. (2023) 186:5620–5637.e16. doi: 10.1016/j.cell.2023.11.006 38065082 PMC10756562

[49] Gubin MM Zhang X Schuster H Caron E Ward JP Noguchi T . Checkpoint blockade cancer immunotherapy targets tumour-specific mutant antigens. Nature. (2014) 515:577–81. doi: 10.1038/nature13988 25428507 PMC4279952

[50] Robbins PF Lu YC El-Gamil M Li YF Gross C Gartner J . Mining exomic sequencing data to identify mutated antigens recognized by adoptively transferred tumor-reactive T cells. Nat Med. (2013) 19:747–52. doi: 10.1038/nm.3161 23644516 PMC3757932

[51] Turajlic S Litchfield K Xu H Rosenthal R McGranahan N Reading JL . Insertion-and-deletion-derived tumour-specific neoantigens and the immunogenic phenotype: a pan-cancer analysis. Lancet Oncol. (2017) 18:1009–21. doi: 10.1016/s1470-2045(17)30516-8 28694034

[52] Comprehensive Molecular Characterization of Human Colon and Rectal Cancer . Comprehensive molecular characterization of human colon and rectal cancer. Nature. (2012) 487:330–7. doi: 10.1038/nature11252 22810696 PMC3401966

[53] Sansregret L Vanhaesebroeck B Swanton C . Determinants and clinical implications of chromosomal instability in cancer. Nat Rev Clin Oncol. (2018) 15:139–50. doi: 10.1038/nrclinonc.2017.198 29297505

[54] Nojadeh JN Behrouz Sharif S Sakhinia E . Microsatellite instability in colorectal cancer. Excli J. (2018) 17:159–68. doi: 10.17179/excli2017-948 29743854 PMC5938532

[55] Han YJ Shao CY Yao Y Zhang Z Fang MZ Gong T . Immunotherapy of microsatellite stable colorectal cancer: resistance mechanisms and treatment strategies. Postgrad Med J. (2024) 100:373–81. doi: 10.1093/postmj/qgad136 38211949

[56] Requesens M Foijer F Nijman HW de Bruyn M . Genomic instability as a driver and suppressor of anti-tumor immunity. Front Immunol. (2024) 15:1462496. doi: 10.3389/fimmu.2024.1462496 39544936 PMC11562473

[57] Kuang X Li J . Chromosome instability and aneuploidy as context-dependent activators or inhibitors of antitumor immunity. Front Immunol. (2022) 13:895961. doi: 10.3389/fimmu.2022.895961 36003402 PMC9393846

[58] Al-Rawi DH Lettera E Li J DiBona M Bakhoum SF . Targeting chromosomal instability in patients with cancer. Nat Rev Clin Oncol. (2024) 21:645–59. doi: 10.1038/s41571-024-00923-w 38992122

[59] Hayes BH Wang M Zhu H Phan SH Dooling LJ Andrechak JC . Chromosomal instability induced in cancer can enhance macrophage-initiated immune responses that include anti-tumor Igg. Elife. (2024) 12. doi: 10.7554/eLife.88054 38805560 PMC11132682

[60] Niu Y Ding C Wang Q Yin J Li L Liu W . Trim6 ablation reverses Icb resistance in Mss gastric cancer by unleashing Cgas-Sting-dependent antitumor immunity. J Exp Clin Cancer Res. (2025) 44:242. doi: 10.1186/s13046-025-03513-5 40817248 PMC12355757

