# Supplementary material for: The chromosomal instability associated gene C2orf76 exerts dual roles in colorectal cancer through pyroptosis and chemotherapy response
Source: Front Oncol. 2026 Aug 12;16:1866584. doi: 10.3389/fonc.2026.1866584 (PMC13506373; doi:10.3389/fonc.2026.1866584)
Supplement: Supplementary file 1 [file DataSheet1.pdf]

Supplementary Material

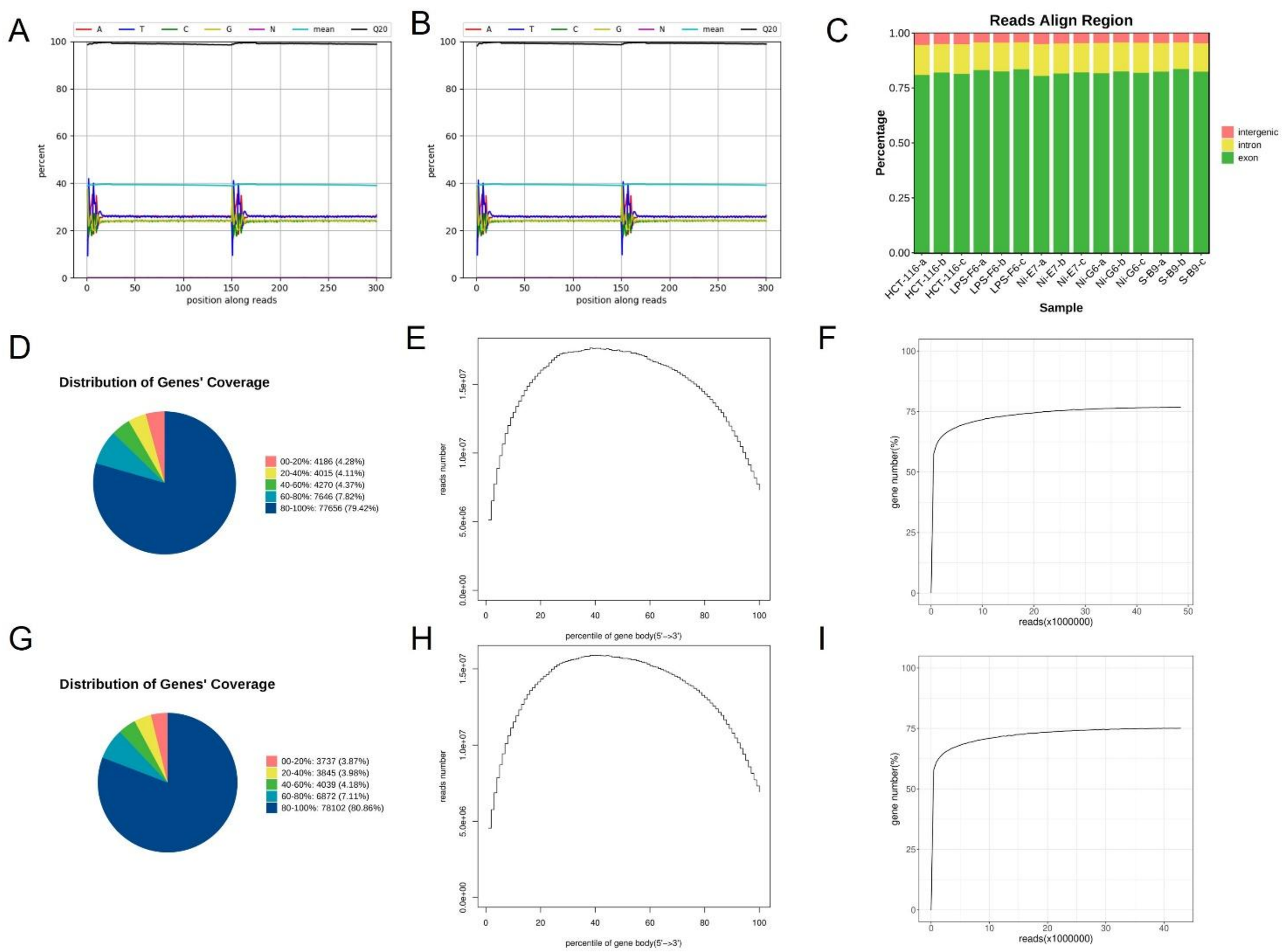

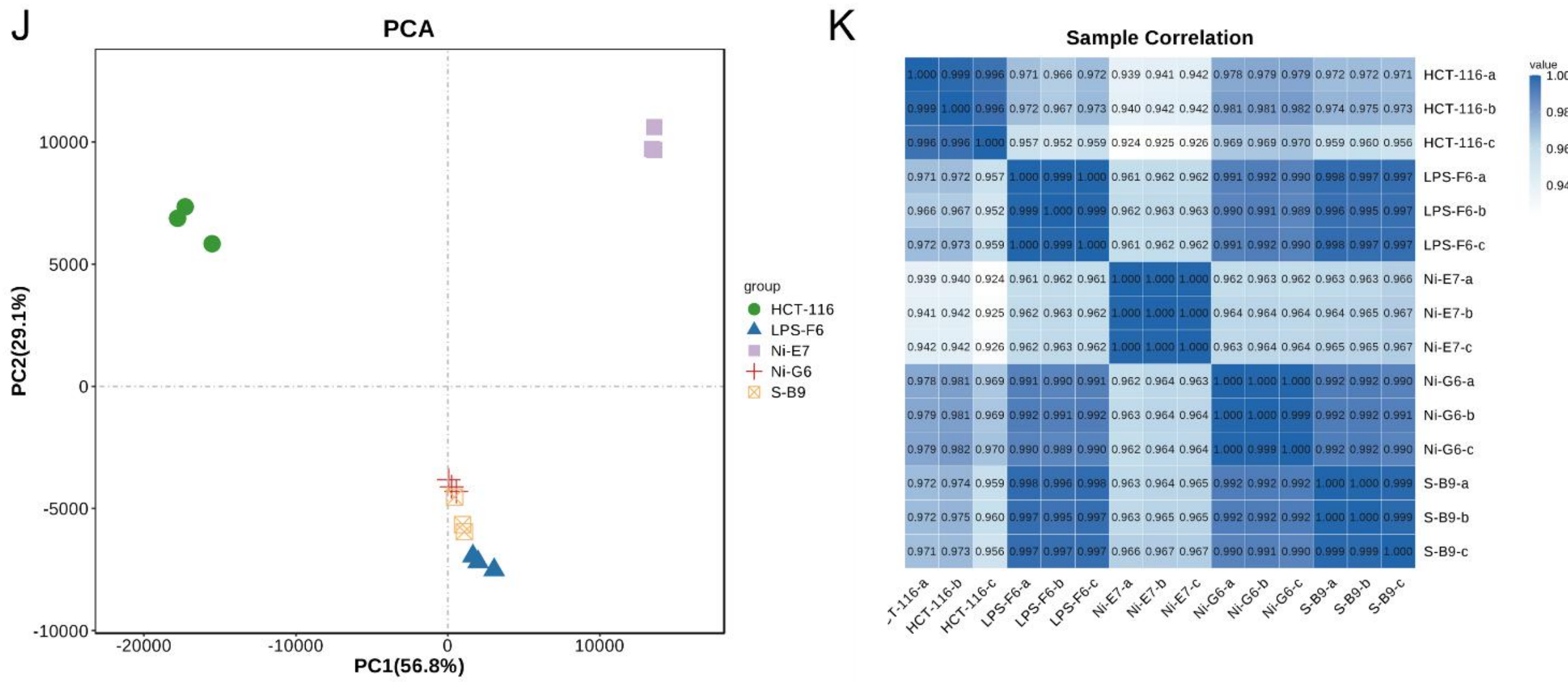

Supplementary Figure 1. Representative images of Quality control of RNA sequencing.

(A, B) Reads status of C2orf76 knockout (Ni-G6) and wild-type (WT) HCT116 cells.

(C) Reads alignment status for all samples; Ni-G6 represents C2orf76 knockout cells, with

n=3 per group.

(D–F) Gene coverage, gene body coverage, and saturation plots for wild-type HCT116 cells.

(G–I) Gene coverage, gene body coverage, and saturation plots for C2orf76 knockout HCT116 cells.

(J) Principal component analysis (PCA) of RNA-seq data from HCT116 control cells (HCT-116) and C2orf76-knockout cells (Ni-G6). Each dot represents an individual biological replicate (n = 3 per group). PC1 and PC2 explain 56.8% and 29.1% of the total variance, respectively. The two groups segregate clearly along PC1, indicating substantial transcriptomic divergence driven by C2orf76 ablation.

(K) Pearson correlation heatmap of all samples based on global gene expression profiles. Samples within the same group (HCT-116 or Ni-G6) exhibit high intra-group correlation coefficients, whereas inter-group correlations are markedly lower, confirming the reproducibility of biological replicates and the robust transcriptional reprogramming induced by C2orf76 knockout. The color scale represents Pearson's correlation coefficient (r) ranging from 0 to 1.

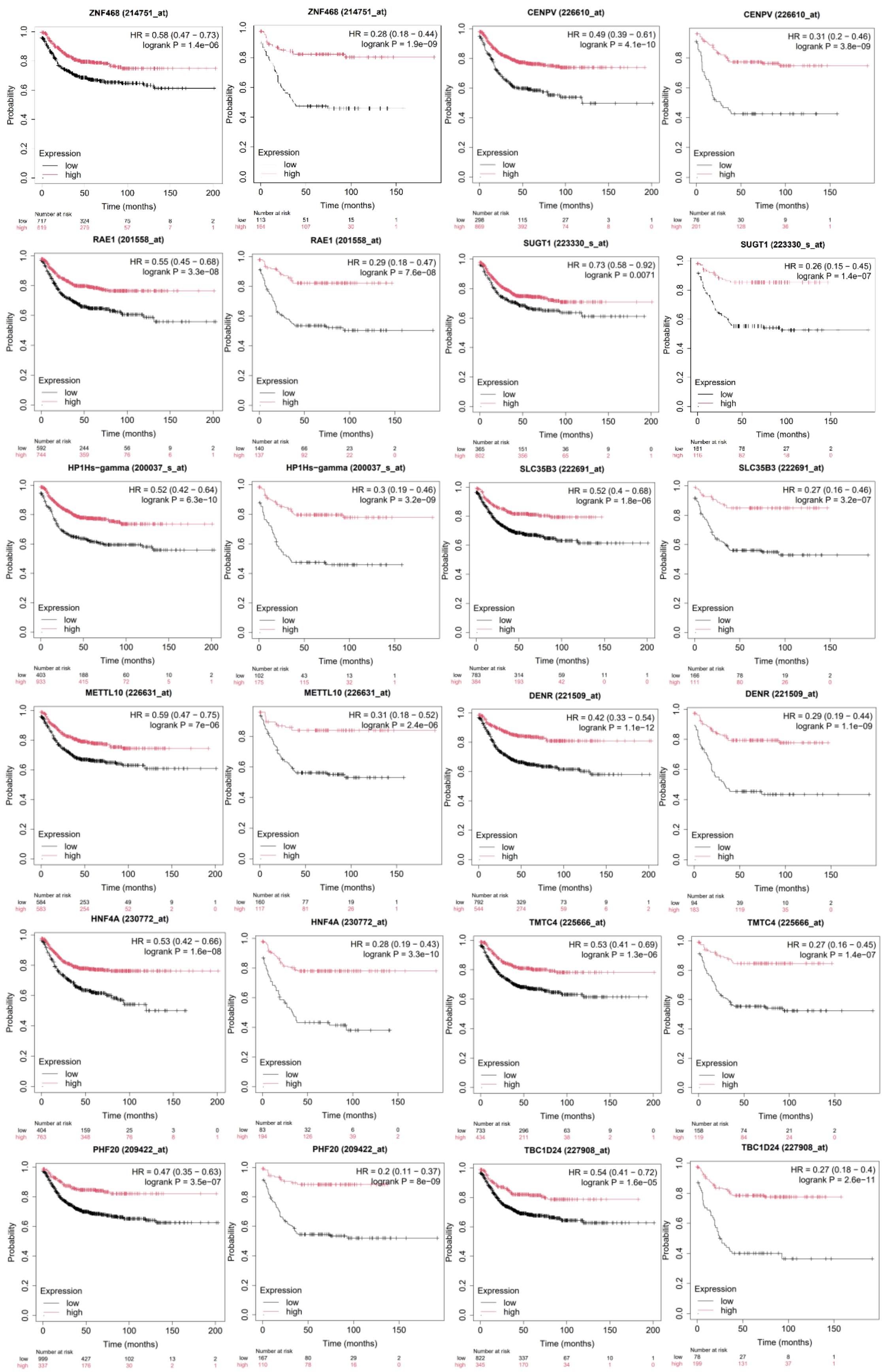
ZNF468 (214751_at)
HR = 0.58 (0.47 − 0.73)
logrank P = 1.4e−06
ZNF468 (214751_at)
HR = 0.28 (0.18 − 0.44)
logrank P = 1.9e−09
CENPV (226610_at)
HR = 0.49 (0.39 − 0.61)
logrank P = 4.1e−10
CENPV (226610_at)
HR = 0.31 (0.2 − 0.46)
logrank P = 3.8e−09
RAE1 (201558_at)
HR = 0.55 (0.45 − 0.68)
logrank P = 3.3e−08
RAE1 (201558_at)
HR = 0.29 (0.18 − 0.47)
logrank P = 7.6e−08
SUGT1 (223330_s_at)
HR = 0.73 (0.58 − 0.92)
logrank P = 0.0071
SUGT1 (223330_s_at)
HR = 0.26 (0.15 − 0.45)
logrank P = 1.4e−07
HP1Hs−gamma (200037_s_at)
HR = 0.52 (0.42 − 0.64)
logrank P = 6.3e−10
HP1Hs−gamma (200037_s_at)
HR = 0.3 (0.19 − 0.46)
logrank P = 3.2e−09
SLC35B3 (222691_at)
HR = 0.52 (0.4 − 0.68)
logrank P = 1.8e−06
SLC35B3 (222691_at)
HR = 0.27 (0.16 − 0.46)
logrank P = 3.2e−07
METTL10 (226631_at)
HR = 0.59 (0.47 − 0.75)
logrank P = 7e−06
METTL10 (226631_at)
HR = 0.31 (0.18 − 0.52)
logrank P = 2.4e−06
DENR (221509_at)
HR = 0.42 (0.33 − 0.54)
logrank P = 1.1e−12
DENR (221509_at)
HR = 0.29 (0.19 − 0.44)
logrank P = 1.1e−09
HNF4A (230772_at)
HR = 0.53 (0.42 − 0.66)
logrank P = 1.6e−08
HNF4A (230772_at)
HR = 0.28 (0.19 − 0.43)
logrank P = 3.3e−10
TMTC4 (225666_at)
HR = 0.53 (0.41 − 0.69)
logrank P = 1.3e−06
TMTC4 (225666_at)
HR = 0.27 (0.16 − 0.45)
logrank P = 1.4e−07
PHF20 (209422_at)
HR = 0.47 (0.35 − 0.63)
logrank P = 3.5e−07
PHF20 (209422_at)
HR = 0.2 (0.11 − 0.37)
logrank P = 8e−09
TBC1D24 (227908_at)
HR = 0.54 (0.41 − 0.72)
logrank P = 1.6e−05
TBC1D24 (227908_at)
HR = 0.27 (0.18 − 0.4)
logrank P = 2.6e−11
Probability
Time (months)
Expression
low
high
Number at risk

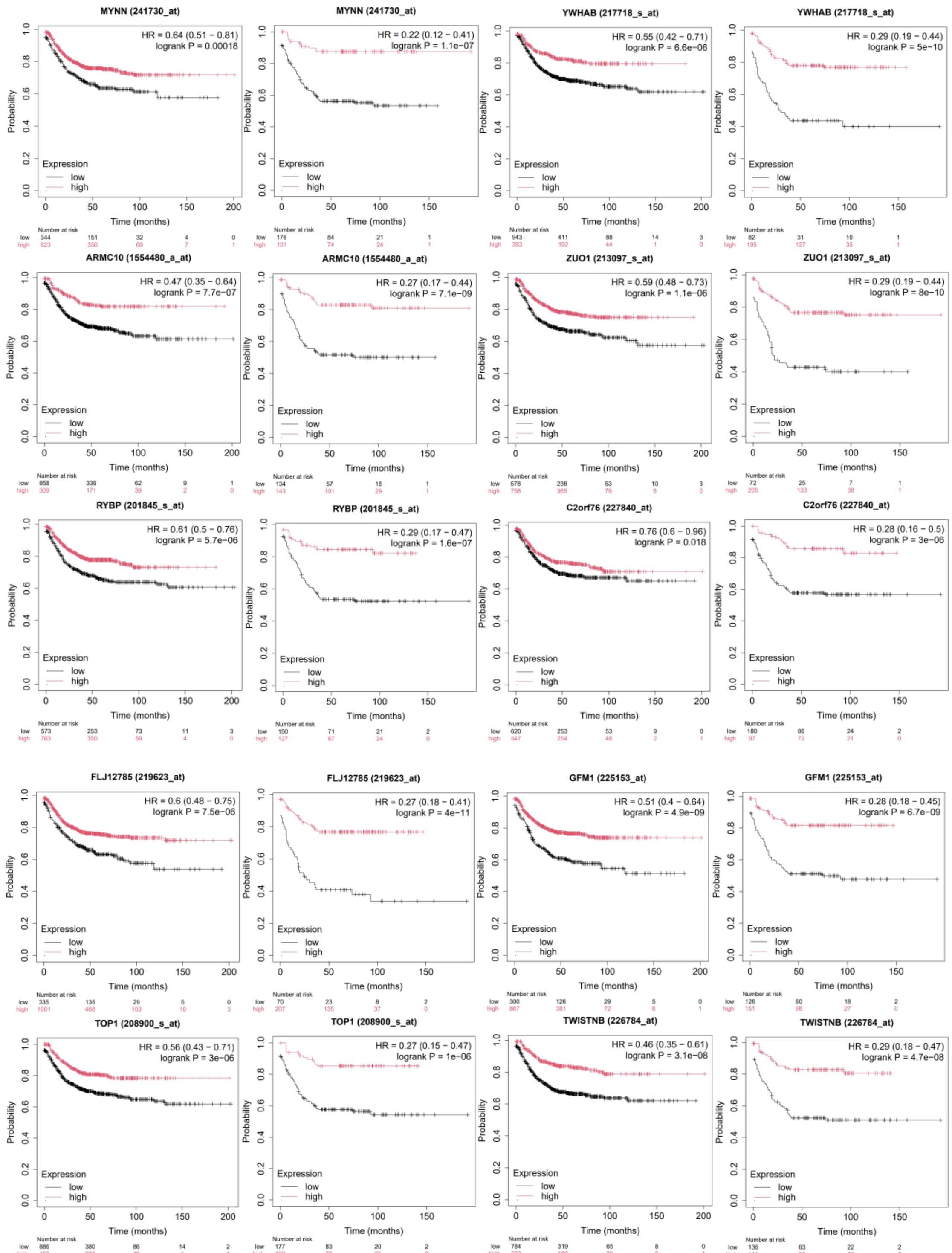

Supplementary Figure 2. Kaplan-Meier analysis (www.kmplot.com) of 22 DEGs in CRC patients with or without TP53 mutation.

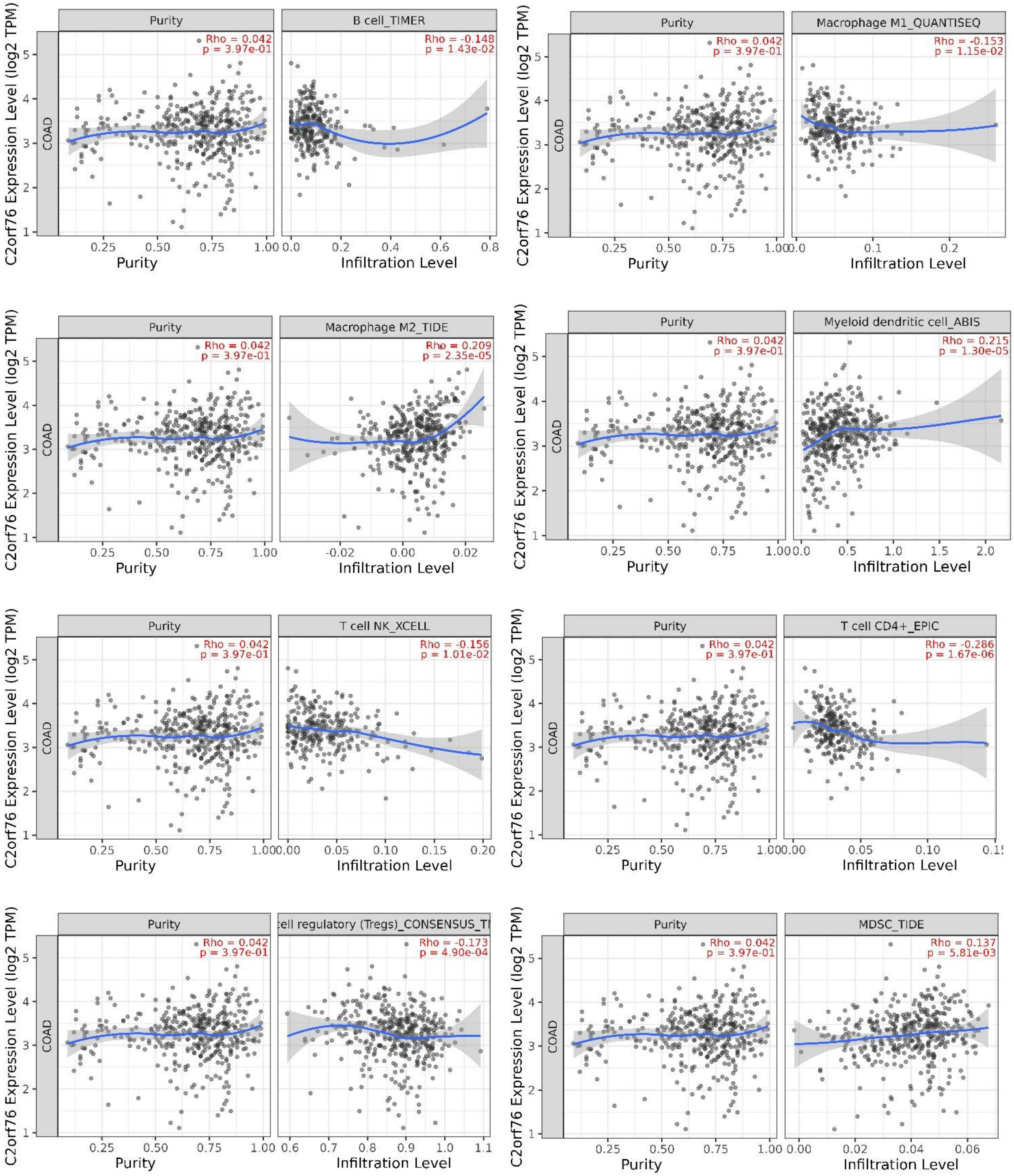

Supplementary Figure 3. Analysis of the correlation between C2orf76 expression and immune cell infiltration in tumors using TIMER 3 database (https://compbio.cn/timer3/).

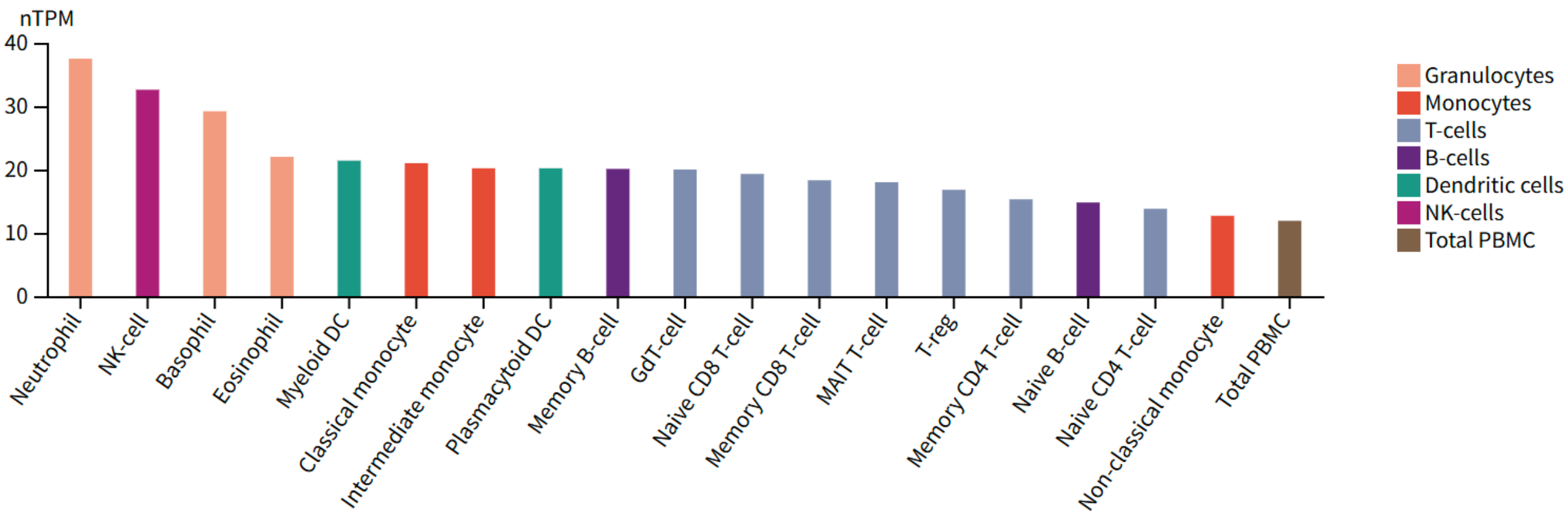

Supplementary Figure 4. Analysis of C2orf76 expression in immune cell through HPA (Human Protein Atlas).

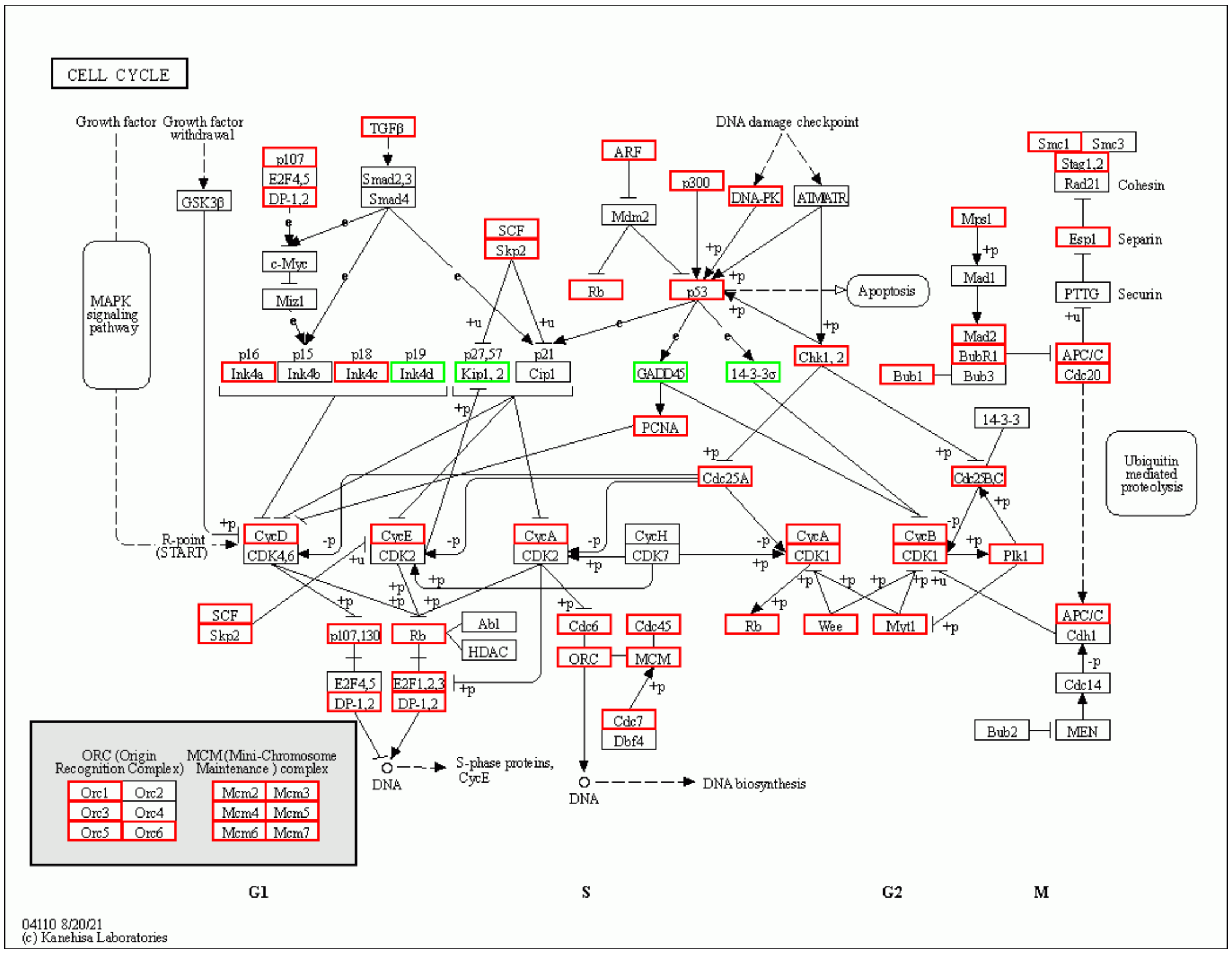
CELL CYCLE
Growth factor
Growth factor withdrawal
GSK3β
MAPK signaling pathway
p107
E2F4,5
DP-1,2
TGFβ
Smad2,3
Smad4
c-Myc
Miz1
SCF
Skp2
ARF
Mdm2
p300
DNA damage checkpoint
DNA-PK
ATM/ATR
Rb
p53
Apoptosis
p16
Ink4a
p15
Ink4b
p18
Ink4c
p19
Ink4d
p27,57
Kip1, 2
p21
Cip1
GADD45
14-3-3σ
Chk1, 2
PCNA
Cdc25A
Smc1
Smc3
Stag1,2
Rad21
Cohesin
Esp1
Separin
PTTG
Securin
Mps1
Mad1
Mad2
BubR1
Bub1
Bub3
APC/C
Cdc20
14-3-3
Cdc25B,C
Ubiquitin mediated proteolysis
R-point (START)
CycD
CDK4,6
CycE
CDK2
CycA
CDK2
CycH
CDK7
CycA
CDK1
CycB
CDK1
Plk1
SCF
Skp2
p107,130
Rb
Abl
HDAC
Cdc6
Cdc45
ORC
MCM
Rb
Wee
Myt1
APC/C
Cdh1
Cdc14
E2F4,5
DP-1,2
E2F1,2,3
DP-1,2
Cdc7
Dbf4
Bub2
MEN
ORC (Origin Recognition Complex)
MCM (Mini-Chromosome Maintenance) complex
Orc1
Orc2
Orc3
Orc4
Orc5
Orc6
Mcm2
Mcm3
Mcm4
Mcm5
Mcm6
Mcm7
DNA
S-phase proteins, CycE
DNA
DNA biosynthesis
G1
S
G2
M
04110 8/20/21
(c) Kanehisa Laboratories

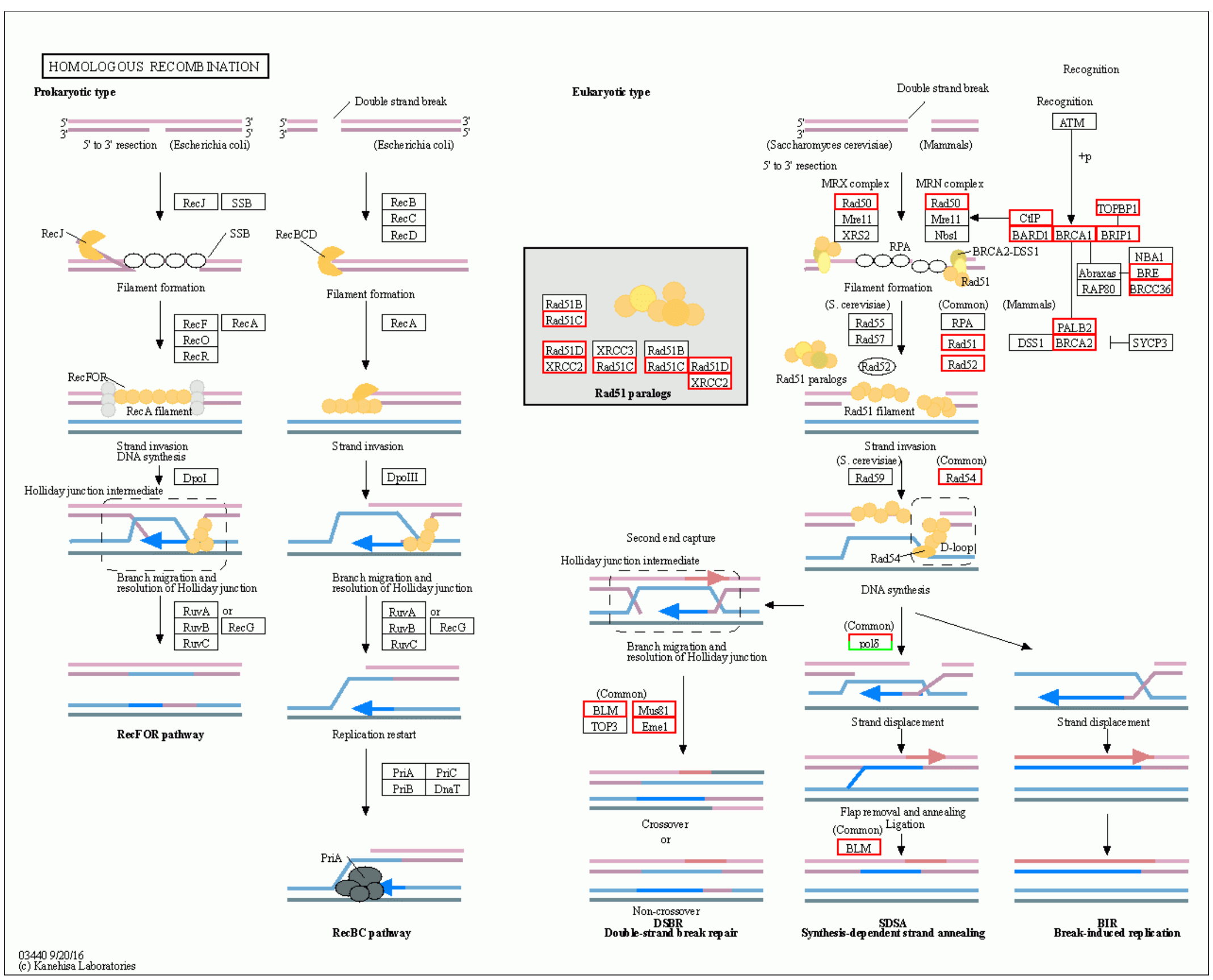
HOMOLOGOUS RECOMBINATION
Prokaryotic type
5' to 3' resection
(Escherichia coli)
Double strand break
(Escherichia coli)
RecJ
SSB
RecB
RecC
RecD
RecJ
SSB
RecBCD
Filament formation
Filament formation
RecF
RecO
RecR
RecA
RecA
RecFOR
RecA filament
Strand invasion DNA synthesis
Strand invasion
DpoI
DpoIII
Holliday junction intermediate
Branch migration and resolution of Holliday junction
Branch migration and resolution of Holliday junction
RuvA
RuvB
RuvC
RecG
RuvA
RuvB
RuvC
RecG
RecFOR pathway
Replication restart
PriA
PriC
PriB
DnaT
PriA
RecBC pathway
Eukaryotic type
Rad51B
Rad51C
Rad51D
XRCC2
XRCC3
Rad51C
Rad51B
Rad51C
Rad51D
XRCC2
Rad51 paralogs
Double strand break
(Saccharomyces cerevisiae)
(Mammals)
5' to 3' resection
Recognition
Recognition
ATM
MRX complex
MRN complex
Rad50
Mre11
XRS2
Rad50
Mre11
Nbs1
CtIP
BARD1
BRCA1
TOPBP1
BRIP1
NBA1
Abraxas
BRE
RAP80
BRCC36
RPA
BRCA2-DSS1
Rad51
Filament formation
(S. cerevisiae)
(Common)
(Mammals)
Rad55
Rad57
RPA
Rad51
Rad52
DSS1
PALB2
BRCA2
SYCP3
Rad52
Rad51 paralogs
Rad51 filament
Strand invasion
(S. cerevisiae)
(Common)
Rad59
Rad54
Rad54
D-loop
Second end capture
Holliday junction intermediate
DNA synthesis
(Common)
polδ
Branch migration and resolution of Holliday junction
(Common)
BLM
Mus81
TOP3
Eme1
Strand displacement
Strand displacement
Crossover
or
Flap removal and annealing
(Common)
Ligation
BLM
Non-crossover
DSBR
Double-strand break repair
SDSA
Synthesis-dependent strand annealing
BIR
Break-induced replication
03440 9/20/16
(c) Kanehisa Laboratories

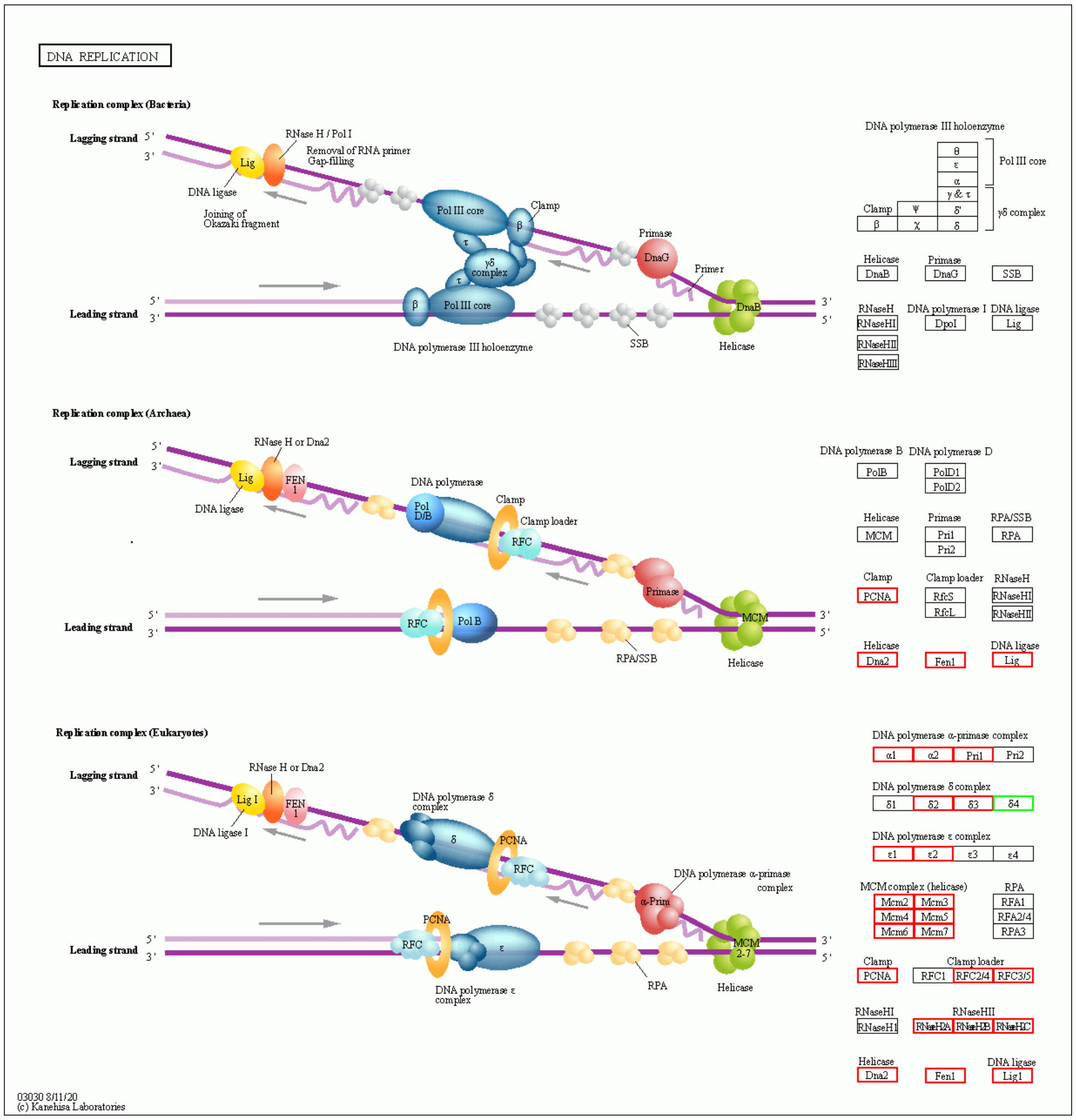
DNA REPLICATION
Replication complex (Bacteria)
Lagging strand
Leading strand
RNase H / Pol I
Removal of RNA primer
Gap-filling
Lig
DNA ligase
Joining of Okazaki fragment
Pol III core
Clamp
γδ complex
Primase
DnaG
Primer
DnaB
DNA polymerase III holoenzyme
SSB
Helicase
DNA polymerase III holoenzyme
Pol III core
γδ complex
Clamp
Helicase
DnaB
Primase
DnaG
SSB
RNaseH
RNaseHI
RNaseHII
RNaseHIII
DNA polymerase I
DpoI
DNA ligase
Lig
Replication complex (Archaea)
RNase H or Dna2
FEN 1
DNA ligase
DNA polymerase
Pol D/B
Clamp
Clamp loader
RFC
Primase
MCM
Pol B
RPA/SSB
Helicase
DNA polymerase B
PolB
DNA polymerase D
PolD1
PolD2
Helicase
MCM
Primase
Pri1
Pri2
RPA/SSB
RPA
Clamp
PCNA
Clamp loader
RfcS
RfcL
RNaseH
RNaseHII
RNaseHIII
Helicase
Dna2
Fen1
DNA ligase
Lig
Replication complex (Eukaryotes)
RNase H or Dna2
Lig I
FEN 1
DNA ligase I
DNA polymerase δ complex
PCNA
RFC
DNA polymerase α-primase complex
α-Prim
MCM 2-7
DNA polymerase ε complex
RPA
Helicase
DNA polymerase α-primase complex
α1
α2
Pri1
Pri2
DNA polymerase δ complex
δ1
δ2
δ3
δ4
DNA polymerase ε complex
ε1
ε2
ε3
ε4
MCM complex (helicase)
Mcm2
Mcm3
Mcm4
Mcm5
Mcm6
Mcm7
RPA
RFA1
RFA2/4
RPA3
Clamp
PCNA
Clamp loader
RFC1
RFC2/4
RFC3/5
RNaseHI
RNaseH1
RNaseHII
RNaseH2A
RNaseH2B
RNaseH2C
Helicase
Dna2
Fen1
DNA ligase
Lig1
03030 8/11/20
(c) Kanehisa Laboratories

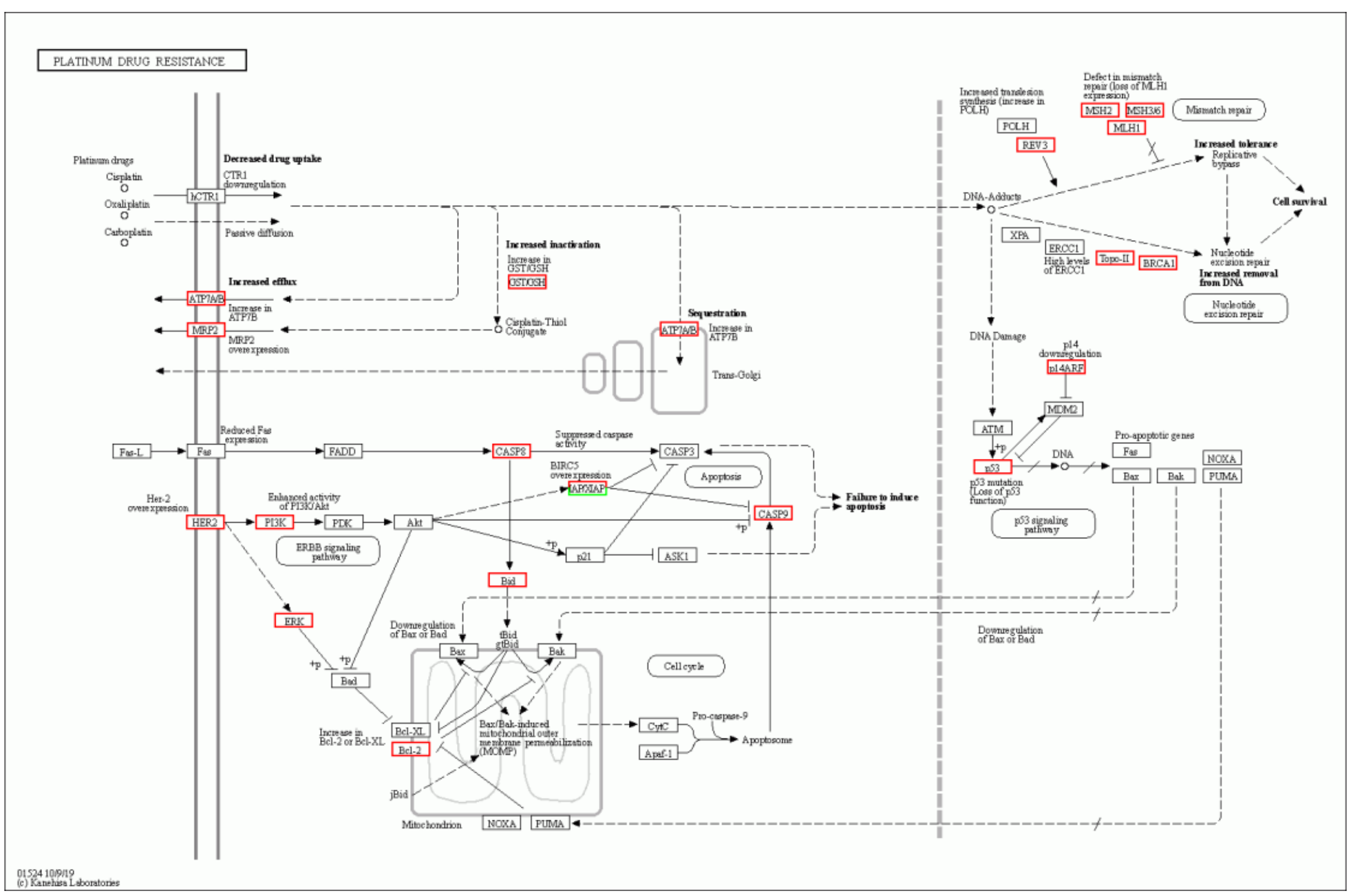
PLATINUM DRUG RESISTANCE
Platinum drugs
Cisplatin
Oxaliplatin
Carboplatin
Decreased drug uptake
CTR1 downregulation
hCTR1
Passive diffusion
Increased efflux
ATP7A/B
Increase in ATP7B
MRP2
MRP2 overexpression
Increased inactivation
Increase in GST/GSH
GST/GSH
Cisplatin-Thiol Conjugate
Sequestration
ATP7A/B
Increase in ATP7B
Trans-Golgi
Fas-L
Fas
Reduced Fas expression
FADD
CASP8
Suppressed caspase activity
CASP3
BIRC5 overexpression
XIAP
Apoptosis
CASP9
Failure to induce apoptosis
Her-2 overexpression
HER2
Enhanced activity of PI3K/Akt
PI3K
PDK
Akt
ERBB signaling pathway
p21
ASK1
Bid
ERK
Bad
Downregulation of Bax or Bad
Bax
tBid
Bak
Increase in Bcl-2 or Bcl-XL
Bcl-XL
Bcl-2
Bax/Bak-induced mitochondrial outer membrane permeabilization (MOMP)
CytC
Apaf-1
Pro-caspase-9
Apoptosome
Cell cycle
Mitochondrion
NOXA
PUMA
Increased translesion synthesis (increase in POLH)
POLH
REV3
Defect in mismatch repair (loss of MLH1 expression)
MSH2
MSH3/6
MLH1
Mismatch repair
Increased tolerance
Replicative bypass
Cell survival
DNA-Adducts
XPA
ERCC1
High levels of ERCC1
Topo-II
BRCA1
Nucleotide excision repair
Increased removal from DNA
Nucleotide excision repair
DNA Damage
p14 downregulation
p14ARF
MDM2
ATM
p53
p53 mutation (Loss of p53 function)
DNA
Pro-apoptotic genes
Fas
Bax
Bak
NOXA
PUMA
p53 signaling pathway
Downregulation of Bax or Bad
01524 10/9/19
(c) Kanehisa Laboratories

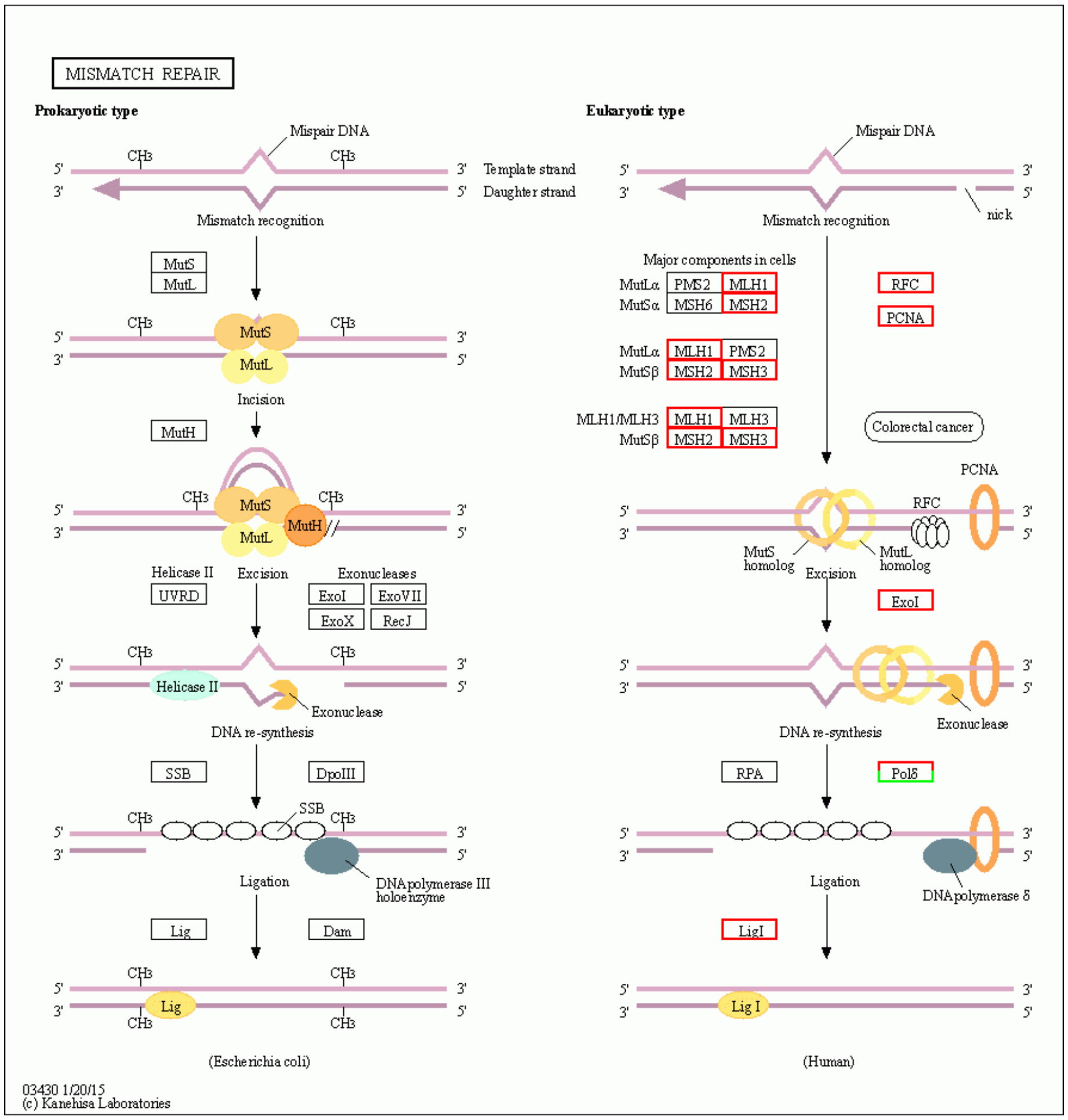

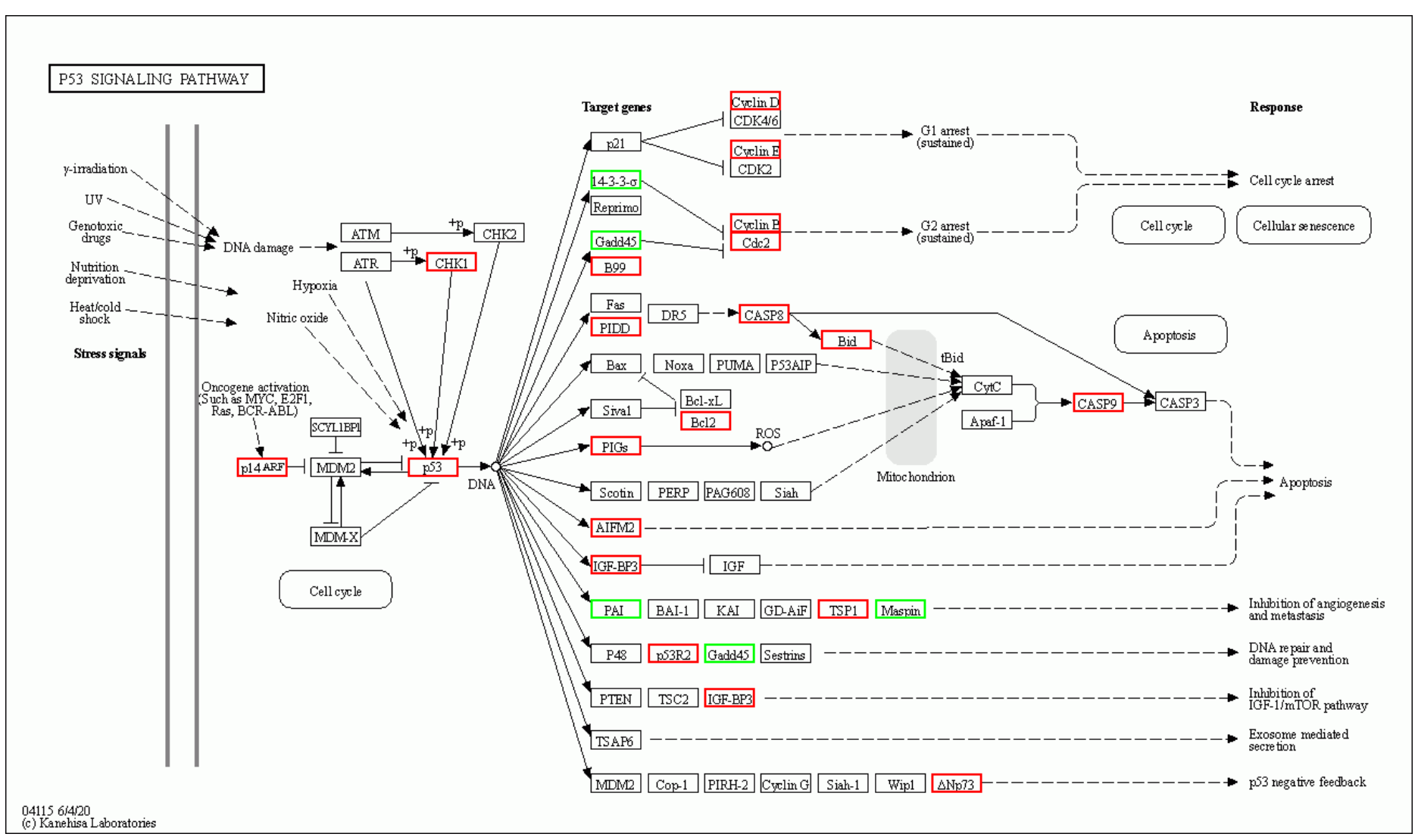

Supplementary Figure 5. KEGG pathway enrichment analysis and mapping of differentially expressed genes (DEGs).

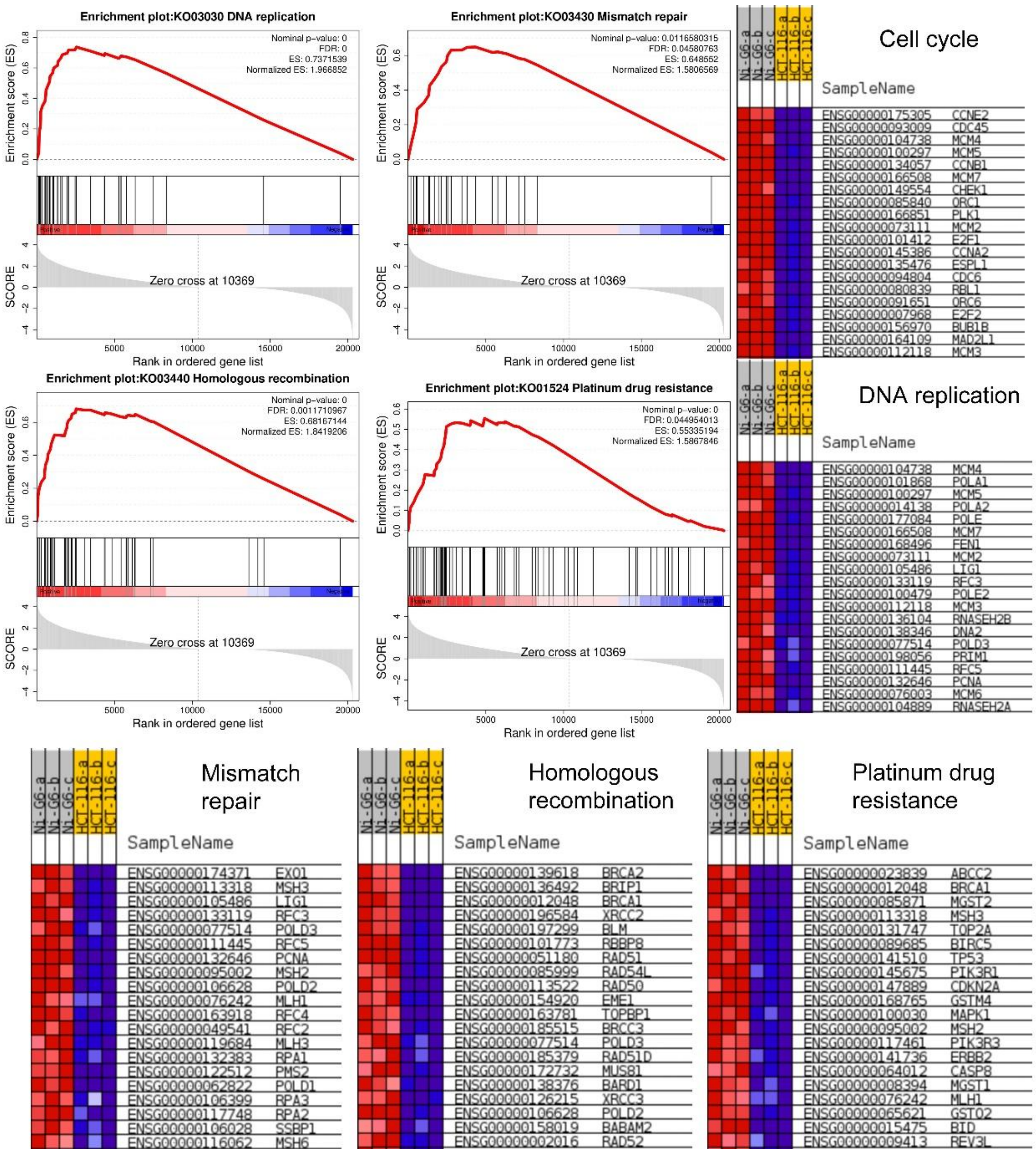

Enrichment plot:KO03030 DNA replication
Nominal p-value: 0
FDR: 0
ES: 0.7371539
Normalized ES: 1.966852
Enrichment score (ES)
SCORE
Zero cross at 10369
Rank in ordered gene list
Enrichment plot:KO03430 Mismatch repair
Nominal p-value: 0.0116580315
FDR: 0.04580763
ES: 0.648552
Normalized ES: 1.5806569
Enrichment score (ES)
SCORE
Zero cross at 10369
Rank in ordered gene list
Enrichment plot:KO03440 Homologous recombination
Nominal p-value: 0
FDR: 0.0011710967
ES: 0.68167144
Normalized ES: 1.8419206
Enrichment score (ES)
SCORE
Zero cross at 10369
Rank in ordered gene list
Enrichment plot:KO01524 Platinum drug resistance
Nominal p-value: 0
FDR: 0.044954013
ES: 0.55335194
Normalized ES: 1.5867846
Enrichment score (ES)
SCORE
Zero cross at 10369
Rank in ordered gene list
Cell cycle
Ni-G6-a
Ni-G6-b
Ni-G6-c
HCT-116-a
HCT-116-b
HCT-116-c
SampleName
ENSG00000175305 CCNE2
ENSG00000093009 CDC45
ENSG00000104738 MCM4
ENSG00000100297 MCM5
ENSG00000134057 CCNB1
ENSG00000166508 MCM7
ENSG00000149554 CHEK1
ENSG00000085840 ORC1
ENSG00000166851 PLK1
ENSG00000073111 MCM2
ENSG00000101412 E2F1
ENSG00000145386 CCNA2
ENSG00000135476 ESPL1
ENSG00000094804 CDC6
ENSG00000080839 RBL1
ENSG00000091651 ORC6
ENSG00000007968 E2F2
ENSG00000156970 BUB1B
ENSG00000164109 MAD2L1
ENSG00000112118 MCM3
DNA replication
SampleName
ENSG00000104738 MCM4
ENSG00000101868 POLA1
ENSG00000100297 MCM5
ENSG00000014138 POLA2
ENSG00000177084 POLE
ENSG00000166508 MCM7
ENSG00000168496 FEN1
ENSG00000073111 MCM2
ENSG00000105486 LIG1
ENSG00000133119 RFC3
ENSG00000100479 POLE2
ENSG00000112118 MCM3
ENSG00000136104 RNASEH2B
ENSG00000138346 DNA2
ENSG00000077514 POLD3
ENSG00000198056 PRIM1
ENSG00000111445 RFC5
ENSG00000132646 PCNA
ENSG00000076003 MCM6
ENSG00000104889 RNASEH2A
Mismatch repair
SampleName
ENSG00000174371 EXO1
ENSG00000113318 MSH3
ENSG00000105486 LIG1
ENSG00000133119 RFC3
ENSG00000077514 POLD3
ENSG00000111445 RFC5
ENSG00000132646 PCNA
ENSG00000095002 MSH2
ENSG00000106628 POLD2
ENSG00000076242 MLH1
ENSG00000163918 RFC4
ENSG00000049541 RFC2
ENSG00000119684 MLH3
ENSG00000132383 RPA1
ENSG00000122512 PMS2
ENSG00000062822 POLD1
ENSG00000106399 RPA3
ENSG00000117748 RPA2
ENSG00000106028 SSBP1
ENSG00000116062 MSH6
Homologous recombination
SampleName
ENSG00000139618 BRCA2
ENSG00000136492 BRIP1
ENSG00000012048 BRCA1
ENSG00000196584 XRCC2
ENSG00000197299 BLM
ENSG00000101773 RBBP8
ENSG00000051180 RAD51
ENSG00000085999 RAD54L
ENSG00000113522 RAD50
ENSG00000154920 EME1
ENSG00000163781 TOPBP1
ENSG00000185515 BRCC3
ENSG00000077514 POLD3
ENSG00000185379 RAD51D
ENSG00000172732 MUS81
ENSG00000138376 BARD1
ENSG00000126215 XRCC3
ENSG00000106628 POLD2
ENSG00000158019 BABAM2
ENSG00000002016 RAD52
Platinum drug resistance
SampleName
ENSG00000023839 ABCC2
ENSG00000012048 BRCA1
ENSG00000085871 MGST2
ENSG00000113318 MSH3
ENSG00000131747 TOP2A
ENSG00000089685 BIRC5
ENSG00000141510 TP53
ENSG00000145675 PIK3R1
ENSG00000147889 CDKN2A
ENSG00000168765 GSTM4
ENSG00000100030 MAPK1
ENSG00000095002 MSH2
ENSG00000117461 PIK3R3
ENSG00000141736 ERBB2
ENSG00000064012 CASP8
ENSG00000008394 MGST1
ENSG00000076242 MLH1
ENSG00000065621 GSTO2
ENSG00000015475 BID
ENSG00000009413 REV3L

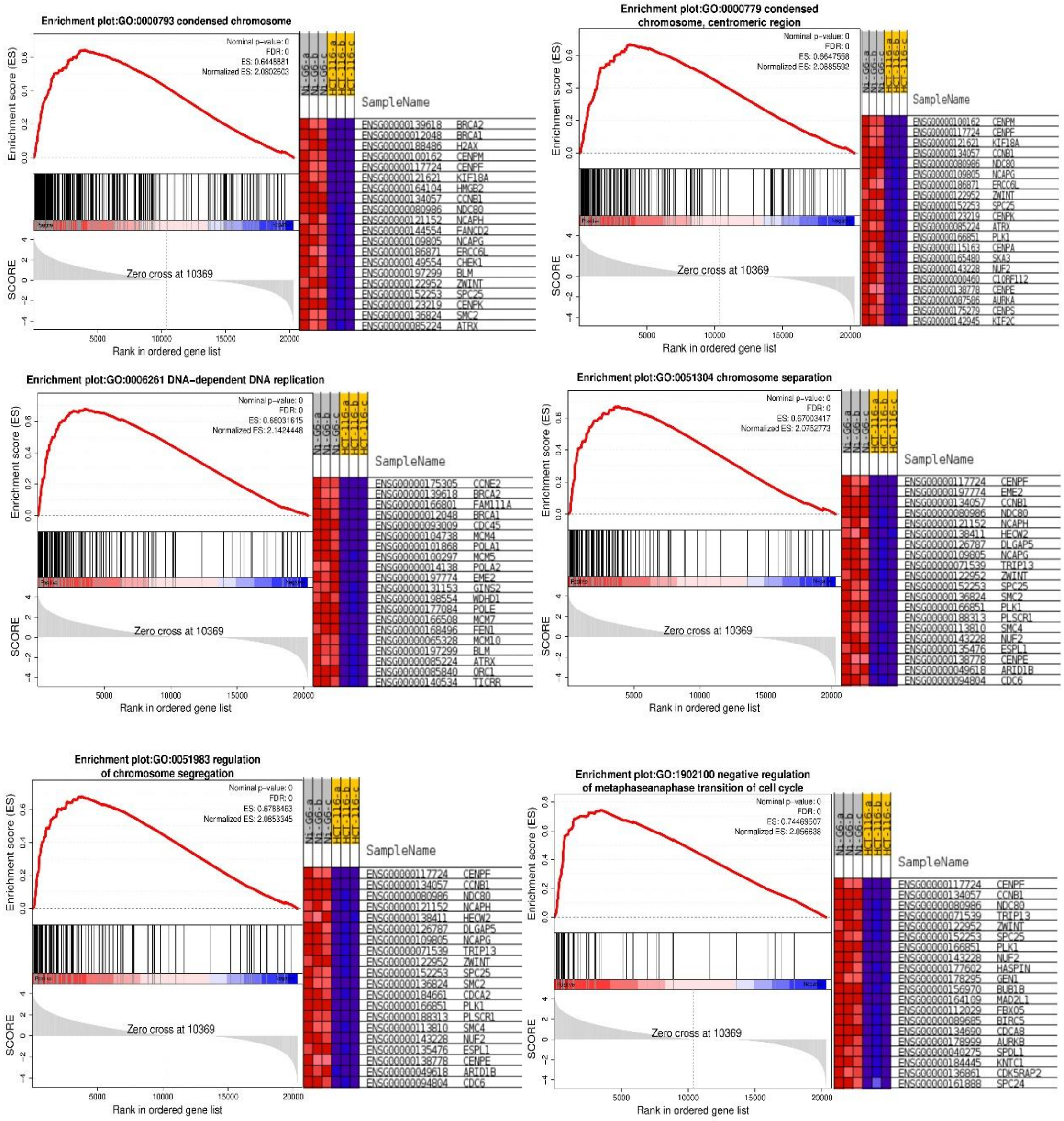

Supplementary Figure 6. Gene Set Enrichment Analysis (GSEA) of gene ontology (GO) terms and KEGG pathways-related gene sets and Top 20 core enrichment genes.

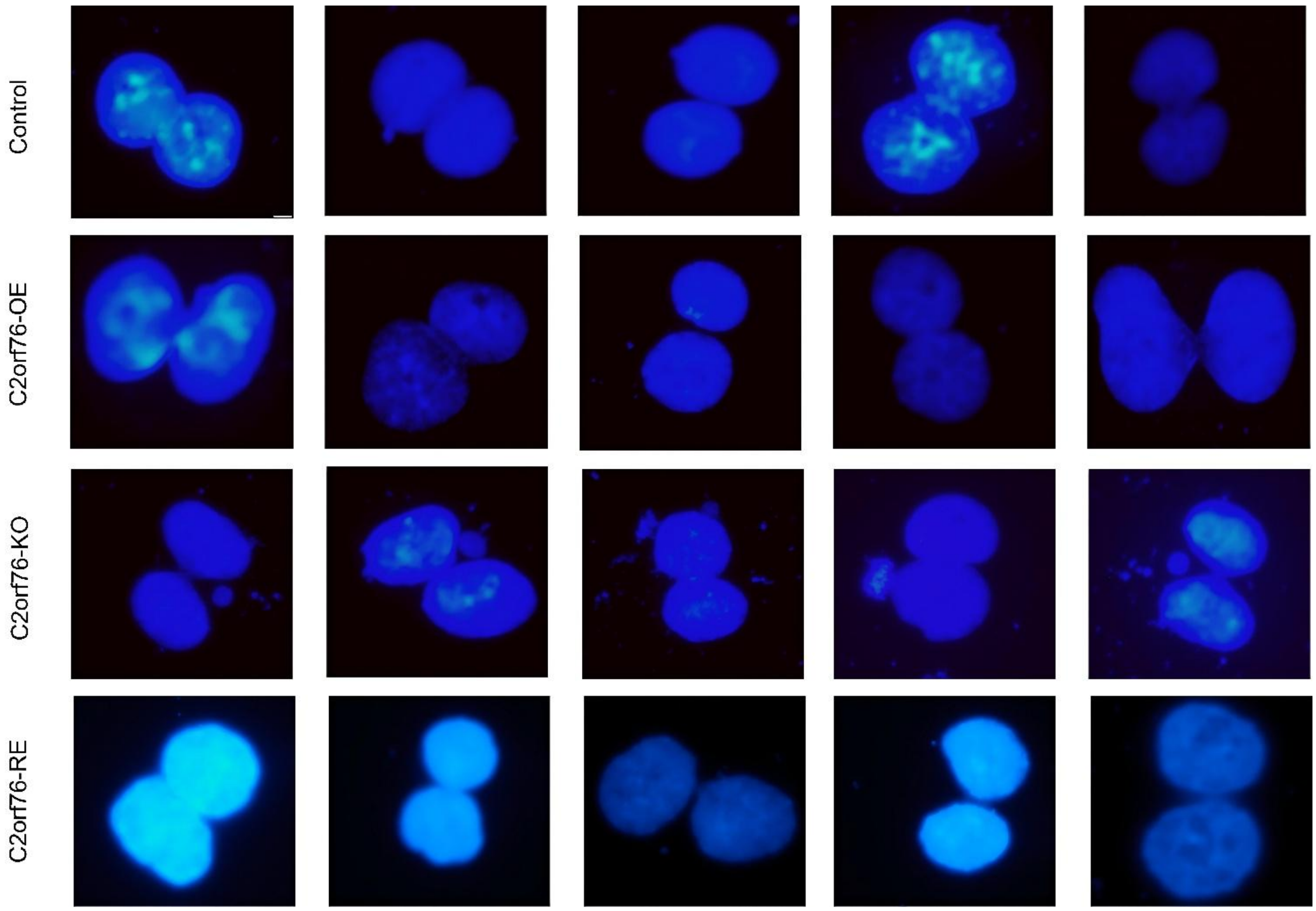

Supplementary Figure 7. Representative images of micronucleus formation in C2orf76 knockout, rescue, and overexpression cells. DAPI staining showed nuclei. Scale bar, 5 μm.
